# Immobilization of trauma patients and in-hospital clinical progression

**DOI:** 10.1590/1980-220X-REEUSP-2025-0552en

**Published:** 2026-08-17

**Authors:** Anne de Aguiar Gondim, Keyliane Costa de Souza, Quíria Ribeiro da Silva Monteiro, Lilia de Souza Nogueira, Antônia Regynara Moreira Rodrigues, Greiciane da Silva Rocha

**Affiliations:** 1 Universidade Federal do Acre, Centro de Ciências da Saúde e do Desporto, Rio Branco, AC, Brazil.; 2 Hospital de Urgência e Emergência de Rio Branco, Secretaria de Saúde do Estado do Acre, Rio Branco, AC, Brazil.; 3 Universidade de São Paulo, Escola de Enfermagem, Departamento de Enfermagem Médico-Cirúrgica, São Paulo, SP, Brazil.

**Keywords:** Prehospital Care, Traumatology, Immobilization, Clinical Evolution, Nursing

## Abstract

**Objective:**

To analyze the association between the immobilization of trauma patients, considering the presence or absence of injury, and in-hospital clinical progression.

**Method:**

A prospective cohort study analyzing trauma patients aged ≥18 who received pre-hospital care and were admitted to a hospital in Acre, Brazil, between September and December 2023. The variables were organized into epidemiological, clinical, care-related, and outcome dimensions. The comparison between the groups (with and without injury) was performed using inferential analysis, with a significance level of 5%.

**Results:**

Immobilized patients with injuries presented greater care complexity, requiring interventions, diagnostic tests, and referrals to specialized units, in addition to longer hospital stays and higher frequency of unfavorable outcomes, such as death. Significant inadequacies were found in immobilization practices, characterized by their application to patients without injury, indicating inconsistencies in clinical decision-making.

**Conclusion:**

Immobilization has been associated with greater complexity of care, but its inadequacy reinforces the need to reorient care practices based on scientific evidence.

## INTRODUCTION

External causes constitute a significant public health problem on a global scale, accounting for a high burden of morbidity, hospitalizations, and preventable mortality. External causes include traffic accidents, assaults, falls, drownings, and other intentional or unintentional injuries, which significantly impact morbidity and mortality indicators, especially among young adults and economically active populations^(1,2)^.

In Brazil, mortality from external causes has shown a growing trend in recent years. In 2022, the national rate reached 61.7 deaths per 100,000 inhabitants, rising to 63.3 in 2023, and reaching 62.8 in 2024. The situation is even more worrying in the Northern Region, where the indicators are higher. In 2024, the state of Acre recorded a rate of 62.0 deaths per 100,000 inhabitants, while the capital, Rio Branco, presented 69.1 deaths per 100,000 inhabitants, highlighting the magnitude of the problem and the need to enhance prevention, surveillance, and organization strategies for the emergency and urgent care network^(3)^.

In addition to mortality, injuries resulting from external causes generate a high demand for health services. In 2025, 1,094,124 hospitalizations from these causes were recorded in Brazil, of which 118,875 occurred in the Northern Region. In the state of Acre, 4,257 hospitalizations were recorded, with 2,703 in Rio Branco, demonstrating the significant hospital demand associated with these conditions. Against this background, it becomes essential to guarantee comprehensive care for trauma patients, encompassing pre-hospital care, hospital care, and rehabilitation efforts^(4)^.

The quality of initial care provided to trauma patients is critical for clinical outcome. This assistance begins at the scene of the traumatic event, with identification of the mechanism of trauma, primary and secondary assessment, stabilization, and safe transport, as recommended by the protocols of Prehospital Trauma Life Support (PHTLS) and of Advanced Trauma Life Support (ATLS)^(5,6)^. In the field of nursing, the following stands out: Advanced Trauma Care for Nurses (ATCN), a program that trains nurses in the systematic assessment and management of trauma patients, strengthening clinical practice and decision-making in patient care^(7)^.

Among the interventions frequently used in the initial management of trauma patients, immobilizations stand out, including cervical spine stabilization and restriction of spinal movement through devices such as cervical collars, rigid boards, and head blocks, as well as immobilization with splints of extremities with suspected fracture(s)^(8)^. Spinal immobilization prevents further injury, while extremity immobilization aims to reduce pain and bleeding, prevent further displacement of bone fragments, and prevent secondary damage to adjacent structures, contributing to greater injury stability and safe transport of the trauma patient^(5)^.

The indication for immobilization in trauma care should be directly related to suspected or confirmed injury, especially in the presence of a risk of spinal instability and/or neurological impairment. International guidelines^(5,6)^ recommend that this decision be based on well-established clinical criteria, such as the mechanism of trauma, physical examination findings, and the presence of a decreased level of consciousness in the trauma patient. However, in clinical practice, unnecessary immobilization is observed in patients with preserved levels of consciousness and no evidence of injury(ies) or significant trauma mechanism, which can contribute to increased discomfort and risk of complications. This condition highlights weaknesses in the clinical decision-making process and reinforces the need for a careful, evidence-based approach that ensures patient safety and the appropriateness of interventions in the context of trauma.

Additionally, contemporary evidence has questioned the universal application of spinal immobilization with rigid devices. In this context, current guidelines propose the Spinal Motion Restriction (SMR), based on selective restriction of spinal movement from an individualized clinical assessment. The indiscriminate use of cervical collars and rigid backboards has been associated with significant adverse effects, including pain and discomfort, increased pressure on soft tissues, risk of pressure injuries, increased intracranial pressure, and impaired respiratory mechanics^(9,10,11)^.

Regarding this topic, care for trauma patients is multidisciplinary, with the nursing team playing a central role in clinical assessment, decision-making, and implementation of stabilization interventions, including orthopedic immobilization procedures, as established by Resolutions No. 713/2022 and No. 705/2022 of the Federal Nursing Council (COFEN)^(12,13)^.

Despite the importance of immobilization for the safety and prognosis of trauma patients, the national literature still presents significant gaps on the subject. There is a lack of evidence that would allow for a more robust understanding of the routine practice of immobilizing trauma patients and its possible relationship with the presence of injury and clinical outcomes. Furthermore, there is a lack of research conducted in the Amazon region, especially in the state of Acre, that addresses this topic and analyzes the role of nursing in the initial and hospital management of trauma patients. Therefore, this study aimed at analyzing the association between the immobilization of trauma patients, considering the presence or absence of injury, and in-hospital clinical progression.

## METHOD

### Ethical Aspects

This research project was analyzed and approved by the Research Ethics Committee of the Universidade Federal do Acre under opinion number 5.870.278 and with certificate of presentation for ethical review (CAAE) number 64424822.3.0000.5020. The research was conducted in full compliance with the ethical precepts established by the Resolutions of the National Health Council, ensuring respect for the principles of human dignity, autonomy, beneficence, non-maleficence, and justice. The secrecy and confidentiality of information were guaranteed, as well as the full protection of participants at all stages of the study.

### Study Design, Period and Local

This is a prospective cohort study, conducted from September to December 2023, at the Emergency and Urgent Care Hospital of Rio Branco (HUERB), Acre, Brazil. HUERB is a large public hospital unit, linked to the Brazilian Public Health System (SUS), which acts as a state reference for urgent and emergency care, covering patients from all municipalities in the state. The institution has a complete healthcare structure (operating room, intensive care unit, observation beds, and specialized inpatient units) to care for patients with clinical, surgical, and traumatic conditions.

### Population or Sample

The study population consisted of trauma patients treated in the trauma emergency department of HUERB. The sample was one of convenience and considered patients aged 18 years or older who suffered blunt, penetrating, or mixed (blunt and penetrating) trauma, received pre-hospital care (PHC), and were admitted to the HUERB emergency room within 24 hours of the traumatic event. Patients readmitted to HUERB due to complications from previous traumatic injuries were excluded.

### Study Protocol

Data collection was carried out daily, from September to December 2023, during day and night shifts, with researchers working in shifts. The information was collected using an instrument previously developed by the researchers that included clinical and epidemiological variables relevant for comparison between groups of immobilized patients with and without injury. The collected data were entered into the REDCap platform, ensuring the security of the information.

The variables analyzed were organized into four main dimensions: epidemiological, clinical, care-related, and outcome-related. The independent variable of the study was the presence of injury(ies) (yes and no) in the patients, which were identified by imaging exams, surgical description, and professional records in the electronic medical record. The variables of the epidemiological dimension included the patients’ sex and age, the characteristics of the trauma, such as external cause (traffic accident, fall, accidental injury, assault and self-inflicted injury), location of occurrence (urban or rural area), and type of trauma (blunt, penetrating or mixed). Subsequently, the interventions (care dimension) performed in pre-hospital and in-hospital care were analyzed, considering the type of immobilization in PHC and the support provided (basic or advanced), as well as respiratory and circulatory support, both in pre-hospital care and in the in-hospital setting.

Indicators of the clinical dimension were also included, comprising variables such as length of stay in the emergency unit and intensive care unit (ICU), mechanical ventilation, and hospital stay, in addition to severity scores Revised Trauma Score (RTS)^(14)^; in pre-hospital and intra-hospital care, the *Rapid* Emergency Medicine Score (REMS)^(15)^ was used and in the intra-hospital environment, the modified REMS (mREMS^(16)^). Additionally, variables related to diagnostic investigation, specialist evaluations, and clinical complications were analyzed, including the performance of imaging exams [chest, pelvis, upper limb, lower limb, and foot x-rays, as well as cervical spine, spine, and abdomen/pelvis computed tomography scans (CT scans)], specialist evaluations (orthopedist, vascular surgeon, and neurosurgeon), and the occurrence of complications such as ventilator-associated pneumonia, pressure injury, surgical site infection, and acute kidney injury.

Finally, the variables of the fourth dimension were considered, including the destination of the traumatized patient after emergency care (hospital discharge, referral to an orthopedic unit, operating room, inpatient unit, intensive care unit, observation unit, elopement, or death) and the final outcome (hospital discharge, elopement, death, transfer to another hospital, discharge at the patient’s request, and other types of discharge). This set of variables enabled a comprehensive analysis of the association between immobilization with and without injury and the trauma profile, care interventions, clinical severity, complexity of care, and outcomes of trauma patients.

The data were collected during the trauma patient’s stay in the emergency room, including clinical management, specialist assessments, tests performed, length of stay in the unit, and destination after treatment. In cases where hospitalization occurred, supplementary information was prospectively obtained from the institutional electronic medical record (G-HOSP system), including diagnosis, ICU admission, occurrence of complications, total length of stay, and final clinical outcome.

### Analysis of Resources and Statistics

Categorical variables were described by absolute (n) and relative (%) frequencies, and numerical variables by measures of central tendency and dispersion. For the comparative analysis between the groups (with and without injury), statistical tests were used according to the nature of the variables and the distribution of the data. The normality of the numerical variables was assessed using the Shapiro-Wilk test and the homogeneity of variances by the Levene test. Given the violation of the assumptions of normality and/or homoscedasticity, non-parametric tests were chosen, including the Wilcoxon-Mann-Whitney test and the Brunner-Munzel test, as appropriate.

The comparison of groups according to categorical variables was performed using Pearson’s chi-square test or Fisher’s exact test. A significance level of 5% (p < 0.05) for all analyses was adopted. Statistical analyses were performed using R software (R Foundation for Statistical Computing, Vienna, Austria), version 4.5.2.

## RESULTS

There were 299 hospital admissions recorded in the trauma emergency unit between September and December 2023. Of these, 162 received PHC, constituting the final sample of the study, and 39 (24.1%) presented injury(ies) identified in imaging exams, surgical description and records in medical charts. In the sample, a predominance of males was observed (68.5%), with a mean and median age of 37.5 (SD 14.3) and 34.5 years, respectively, and a range of 19 to 91 years. There was no significant difference between patients with and without injury in relation to sex and age (p > 0.05).

The distribution of patients according to the characteristics of the trauma showed that traffic accidents were the main external cause in both groups, with a higher proportion among patients with injury (76.9%) compared to those without injury (57.8%). There was no significant difference between the groups with respect to the external cause (p = 0.274). Regarding the location of the trauma, a predominance of events in urban areas was observed, both among patients without injury (82.1%) and among those with injury (84.6%), with no significant association identified in this comparative analysis (p = 0.720) (Table 1).

**Table 1:** Patients (n = 162) according to presence of injury and characteristics of trauma – Rio Branco, AC, Brazil, 2023.

Variables	Presence of injury		p-value
	No	Yes	
	n (%)	n (%)	
External cause			
Traffic accident	71 (57.7)	30 (76.9)	
Fall	19 (15.5)	2 (5.1)	0.274*
Accidental injury	5 (4.1)	2 (5.1)	
Aggression	26 (21.1)	5 (12.9)	
Self-inflicted injury	2 (1.6)	-	
Location of the incident			
Urban area	101 (82.1)	33 (84.6)	0.720**
Rural area	22 (17.9)	6 (15.4)	
Type of trauma			
Blunt	80 (65.1)	18 (46.2)	< 0.001*
Mixed	19 (15.4)	19 (48.7)	
Penetrating	24 (19.6)	2 (5.1)	

*Fisher’s exact test;

**Pearson’s chi-square test.

Conversely, Table 1 also shows that the type of trauma demonstrated a statistically significant association between patients with injury (p < 0.001), with blunt trauma being more frequent among those without injury (65.1%) and mixed trauma (blunt and penetrating) with injury (48.7%). Isolated penetrating trauma, in turn, was more frequent in the group without injury (19.6%).

Regarding the care dimension (Table 2), an association was observed between patients with and without injury and the care interventions in PHC: immobilization (p < 0.001), basic circulatory support (p = 0.014), and advanced circulatory support (p = 0.005). All patients with injury underwent immobilization, and among those without injury, 56.9% were also immobilized. Furthermore, basic circulatory support performed in PHC was more frequent among patients with injury (41.0%) compared to those without injury (21.1%). Similarly, advanced circulatory support, also performed in the pre-hospital setting, was more frequent among patients with injury (89.7%), highlighting a greater need for hemodynamic interventions in this group. There was no significant difference between the groups (p > 0.050) in relation to respiratory care and support (basic or advanced) in the pre-hospital setting and to respiratory and circulatory support in the in-hospital setting.

**Table 2:** Patients (n = 162) according to presence of injury and care interventions in pre- and intra-hospital care – Rio Branco, AC, Brazil, 2023.

Variables	Presence of injury		p-value
	No	Yes	
	n (%)	n (%)	
Immobilization in PHC			
No	53 (43.1)	-	<0.001*
Yes	70 (56.9)	39 (100.0)	
Type of support			
Basic	97 (78.9)	32 (82.1)	0.668**
Advanced	26 (21.1)	7 (17.9)	
Basic respiratory support in PHC			
No	112 (91.1)	36 (92.3)	0.809**
Yes	11 (8.9)	3 (7.7)	
Advanced respiratory support in PHC			
No	117 (95.1)	37 (94.9)	0.950**
Yes	6 (4.9)	2 (5.1)	
Basic respiratory support in PHC			
No	97 (78.9)	23 (59.0)	0.014**
Yes	26 (21.1)	16 (41.0)	
Advanced circulatory support in PHC			
No	41 (33.3)	4 (10.3)	0.005**
Yes	82 (66.7)	35 (89.7)	
In-hospital respiratory support			
No	109 (88.6)	35 (92.1)	0.542**
Yes	14 (11.4)	3 (7.9)	
In-hospital circulatory support			
No	53 (43.1)	17 (43.6)	0.956**
Yes	70 (56.9)	22 (56.4)	

*Fisher’s exact test;

**Pearson’s chi-square test.

Legend: PHC – Pre-Hospital Care.

Data in Table 3 show that the groups were similar in terms of trauma severity as measured by the RTS index in the pre-hospital care (p = 0.480), and RTS (p = 0.404), REMS (p = 0.607) and mREMS (p = 0.839) in the intra-hospital care, as well as the length of stay in the emergency unit (p = 0.265) and in the ICU (p = 0.561), and the use of mechanical ventilation (p = 0.108). In contrast, the length of hospital stay showed a statistically significant difference between the groups (p < 0.001), being higher among immobilized patients with injury (median of 3 days) compared to those without injury (0 days).

**Table 3:** Descriptive statistics of clinical dimension indicators according to the presence of injury – Rio Branco, AC, Brazil, 2023 (n = 162).

Variables	Presence of injury		p
	No	Yes	
	Median (Q1–Q3)		
Time in the emergency unit (in hours)	2.6 (1.5 – 4.7)	2.0 (1.1 – 4.5)	0.265*
Time in the ICU (in days)	14 (6 – 19)	14 (13.2 – 17.2)	0.561*
Mechanical ventilation time (in days)	4 (3 – 6.5)	11 (9.5 – 12.5)	0.108*
Length of hospital stay	0 (0 – 1)	3 (2 – 5)	<0.001**
RTS - PHC	12 (12)	12 (12)	0.480*
RTS – Intra-hospital	7.8 (7.8 – 7.8)	7.8 (7.8 – 7.8)	0.404*
REMS – Intra-hospital	2 (0 – 4)	2 (0 – 3)	0.697*
mREMS – Intra-hospital	1 (0 – 2)	1 (0.2)	0.839*

*Wilcoxon-Mann-Whitney test;

**Brunner-Munzel test.

Legend: ICU – Intensive care unit; RTS – Revised Trauma Score; PHC – Pre-Hospital Care; REMS – Rapid Emergency Medicine Score; mREMS – modified Rapid Emergency Medicine Score.

Analysis of the association between the presence or absence of injury and diagnostic investigations, specialist evaluations, and clinical complications revealed significant differences in some specific variables (Table 4). Regarding imaging exams, upper limb radiography was more frequent among immobilized patients with injury (51.3%) compared to those without injury (13.8%) (p < 0.001). Similarly, lower limb radiography was also significantly more frequently performed in the injury group (69.2%) (p < 0.001), suggesting a higher incidence of orthopedic injuries in these patients. With regard to specialist assessments, the orthopedist’s assessment (p < 0.001) was more frequent among immobilized patients with injury (87.1%) compared to those without injury (27.6%).

**Table 4:** Patients (n = 162) according to the presence of injury and diagnostic tests, specialist evaluations, and clinical complications – Rio Branco, AC, Brazil, 2023.

Variables	Presence of injury		p-value
	No	Yes	
	n (%)	n (%)	
Chest X-ray			
No	53 (43.1)	14 (35.9)	0.428*
Yes	70 (56.9)	25 (64.1)	
Pelvic X-ray			
No	72 (58.5)	19 (48.7)	0.283*
Yes	51 (41.5)	20 (51.3)	
UL X-ray			
No	106 (86.2)	19 (48.7)	<0.001*
Yes	17 (13.8)	20 (51.3)	
LL X-ray			
No	90 (73.2)	12 (30.8)	<0.001*
Yes	33 (26.8)	27 (69.2)	
Cervical CT scan			
No	95 (77.2)	31 (79.5)	0.769*
Yes	28 (22.8)	8 (20.5)	
Spinal tomography			
No	99 (80.5)	33 (84.6)	0.564*
Yes	24 (19.5)	6 (15.4)	
Orthopedist’s Evaluation			
No	89 (72.4)	5 (12.8)	<0.001*
Yes	34 (27.6)	34 (87.1)	
Vascular Surgeon Assessment			
No	120 (97.6)	39 (100.0)	1.000**
Yes	3 (2.4)	-	
Neurosurgeon’s Assessment			
No	75 (61.0)	26 (66.7)	0.524*
Yes	48 (39.0)	13 (33.3)	
ICU admission			
No	116 (94.3)	35 (89.7)	0.325*
Yes	7 (5.7)	4 (10.3)	
Ventilator-associated pneumonia			
No	122 (99.2)	37 (94.9)	0.144**
Yes	1 (0.8)	2 (5.1)	
Pressure ulcer			
No	122 (99.2)	37 (94.9)	0.144**
Yes	1 (0.8)	2 (5.1)	
Surgical site infection			
No	122 (99.2)	37 (94.9)	0.144**
Yes	1 (0.8)	2 (5.1)	
Acute kidney injury			
No	122 (99.2)	37 (94.9)	0.144**
Yes	1 (0.8)	2 (5.1)	

*Pearson’s chi-square test;

**Fisher’s exact test.

Legend: UL – Upper limbs; LL – Lower limbs; ICU – Intensive care unit; MV – Mechanical ventilation.

Concerning clinical complications (ventilator-associated pneumonia, pressure injury, surgical site infection, and acute kidney injury), no statistically significant associations were identified between the groups (p = 0.144). Overall, these results highlight that immobilized patients who presented with injuries had conditions associated with the musculoskeletal system, such as a higher frequency of radiographic examinations of limbs and orthopedic evaluation (Table 4).

Data in Table 5 show a higher frequency of hospital discharge after emergency room care among patients without injury (68.4%) compared to those with injury (10.3%). Conversely, immobilized patients with injuries were more frequently referred to units with greater care complexity, notably the orthopedic unit (33.3%), the operating room (23.1%), and inpatient units (23.1%), highlighting differences within the groups (p < 0.001).

**Table 5:** Patients (n = 162) according to the presence of injury and destination after emergency care and final outcome – Rio Branco, AC, Brazil, 2023.

Variables	Presence of injury		p*
	No	Yes	
	n (%)	n (%)	
Destination after emergency			
Hospital discharge	84 (68.4)	4 (10.3)	
Orthopedic Unit	11 (8.9)	13 (33.3)	<0.001
Operating Room	12 (9.8)	9 (23.1)	
Inpatient unit	9 (7.3)	9 (23.1)	
Intensive care unit	4 (3.2)	3 (7.7)	
Elopement	1 (0.8)	-	
Observation unit	1 (0.8)	-	
Death	-	1 (2.5)	
Other	1 (0.8)	-	
Final outcome			
Hospital discharge	109 (94.8)	32 (84.2)	
Elopement	3 (2.6)	1 (2.6)	
Death	2 (1.7)	3 (7.9)	0.044
Other types of discharge	1 (0.9)	-	
Transfer to another hospital	-	1 (2.6)	
Discharge on request	-	1 (2.6)	

*Fisher’s exact test.

In respect to the outcome of hospitalization (Table 5), a statistically significant association (p = 0.044) was also observed among the groups analyzed in the study. Hospital discharge was more frequent among patients without injury (94.8%), and death was more frequent among those with injury (7.9%). In general, these findings indicate that immobilized trauma patients with injuries present with high levels of care complexity and severity, evidenced by referrals to specialized sectors after emergency room care and a higher incidence of unfavorable outcomes, such as death.

## DISCUSSION

The results of this study show that immobilization in the presence of injury is directly related to the characteristics of the trauma, the care profile in pre-hospital care, and the patients’ clinical progression. Regarding the mechanism of trauma, it is observed that higher-energy events, such as traffic accidents, were more frequently associated with immobilization resulting in injury, corroborating the findings of a previous study^(17)^ that indicates these events as the main causes of serious and potentially disabling injuries. Furthermore, the higher frequency of indication in traumas with mixed components (blunt/penetrating) reinforces that the complexity of the injury mechanism directly influences the clinical decision, as discussed in the guidelines of the American College of Surgeons^(18)^, which emphasize the mechanism of trauma as one of the cornerstones of the initial assessment.

In the context of PHC, the results demonstrate partial consistency (more than 50% of the patients without injury were immobilized) between the clinical assessment and the immobilization procedure, indicating that this intervention remains strongly incorporated into clinical practice. The occurrence of immobilization in patients without a clear clinical indication highlights the persistence of a model based on universal precaution. This finding is consistent with the contemporary literature^(18,19)^, which points to the immobilization that is frequently and broadly applied, even in the absence of robust evidence to support its indiscriminate use. Recent guidelines have recommended transitioning to strategies based on the concept of SMR, which guides the selective restriction of spinal movement based on well-defined clinical criteria^(9)^.

Within this framework, when it is not possible to safely rule out the presence of injury, especially in scenarios of limited assessment in PHC, decreased level of consciousness, and intoxication, movement restriction may be indicated as a precautionary measure. However, the indiscriminate use of rigid devices, such as the spine board, should be avoided, prioritizing safer, more comfortable, and individualized approaches. Thus, current practice does not support routine immobilization in the face of uncertainty, but rather a structured clinical decision-making process that balances risks and benefits for the patient^(9)^.

Additionally, the observed association between the presence of injury and a greater need for circulatory support in PHC suggests that these patients present with greater physiological and hemodynamic impairment. This finding reinforces the relationship between the indication for immobilization and the clinical severity of the trauma patient, showing that the intervention has been used more frequently in scenarios of greater care complexity. This result is consistent with the literature, which demonstrates that more advanced interventions in the pre-hospital setting tend to focus on patients with greater trauma severity and a higher risk of clinical instability^(20)^.

Thus, immobilization in this context can be interpreted as an indirect marker of severity, reflecting more the clinical perception of risk by healthcare teams than an intervention that alone determines outcomes. This interpretation reinforces the idea that its indication tends to occur in patients with greater physiological impairment and potential for instability. In line with these findings, the literature indicates that, in the pre-hospital setting, the nursing team plays a central role in the hemodynamic and clinical stabilization of the trauma patient, acting in circulatory and respiratory support through interventions such as venipuncture, medication administration, continuous monitoring of vital signs, provision of oxygen therapy, and wound care^(21)^. In light of this, immobilization should be understood as part of an integrated set of care measures, which also includes maintaining tissue perfusion and preventing secondary complications.

Regarding clinical progression, it was found that immobilized patients with injuries had longer hospital stays, which reinforces its association with greater complexity of care. Although severity scores did not show significant differences between the groups, this finding suggests that initial clinical assessment and professional judgment can identify dimensions of severity not fully measured by physiological scales. This phenomenon is discussed by researchers^(22)^, who emphasize that decision-making in trauma is multifactorial, involving not only objective parameters, but also the mechanism of trauma, the context of care, and the professional’s experience.

In the in-hospital setting, immobilized patients with injuries were more frequently subjected to diagnostic tests and specialized assessments, especially in the orthopedic area. This result reinforces the association between adequate immobilization and suspected structural injuries^(23)^. The increased use of imaging exams in these patients may reflect both the greater severity of the condition and the need for diagnostic confirmation in the face of possible hidden injuries, underscoring a more cautious approach by healthcare teams.

Regarding the destination after emergency room care and hospital outcomes, it was observed that immobilized patients with injuries had a greater need for hospitalization in specialized units, a higher frequency of surgical interventions, and a higher incidence of death. These findings reinforce the idea that immobilization is strongly associated with patients presenting with greater severity and clinical complexity^(18,24)^. However, it is crucial to emphasize that this association should not be interpreted as a causal relationship. Recent evidence^(25,26)^ indicates that pre-hospital immobilization is not consistently associated with a reduction in neurological deficits or an improvement in clinical outcomes. Therefore, immobilization should be understood as part of a set of interventions aimed at more severely ill patients, and not as an isolated factor determining clinical outcomes.

Given this scenario, the need to reassess care practices related to immobilization in trauma becomes evident. The literature points to a transition from a model based on universal immobilization to a more selective approach, grounded in the concept of SMR*.* As highlighted in studies and guidelines^(9,27)^, this approach allows for the reduction of unnecessary interventions, minimizes associated risks, and maintains patient safety, promoting greater rationality in care.

Thus, the results of this study not only reinforce the association between immobilization and greater clinical severity, but also highlight gaps in the appropriateness of its indication, emphasizing the importance of incorporating classic evidence-based protocols, such as the National Emergency X-Radiography Utilization Study (NEXUS) and Canadian C-Spine Rule^(28,29)^ criteria. The adoption of these strategies, combined with the continuous training of healthcare teams, especially nursing staff, who occupy a strategic position in the evaluation and execution of these interventions, can contribute to safer, more efficient care aligned with the best available scientific evidence.

Additionally, regarding limb immobilization and pelvic stabilization, it is observed that these interventions have received greater support in recent literature when compared to routine spinal immobilization. Studies indicate that extremity immobilization, especially in cases of suspected or confirmed fractures, contributes to pain reduction, prevention of further soft tissue injuries, and decreased risk of complications during patient transport^(30)^. Similarly, early pelvic stabilization, through the use of pelvic binders, has been recommended in patients with suspected unstable pelvic fractures, and is associated with a reduction in pelvic volume, control of bleeding, and improvement in hemodynamic stability^(30)^.

Guidelines from the World Society of Emergency Surgery emphasize that the early application of pelvic stabilization devices in pre-hospital care and in the emergency room is an important measure in the initial management of severe pelvic trauma^(30)^. Thus, unlike indiscriminate cervical immobilization, limb immobilization and pelvic stabilization have more well-established and evidence-supported indications, and should be incorporated judiciously and early into care protocols, contributing to the reduction of complications and the improvement of clinical outcomes.

In summary, the findings of this study demonstrate that immobilization with injury is associated with a patient profile requiring more complex care in the context of trauma, evidenced by a higher frequency of combined trauma, a greater need for circulatory interventions in PHC and for diagnostic tests focused on the musculoskeletal system, a greater demand for orthopedic evaluation, and referral to units with higher technological density, such as operating rooms and hospital inpatient care. Additionally, these patients had longer hospital stays and a higher frequency of hospital deaths, indicating that the adequacy of immobilization is more related to the burden of injuries and the complexity of care than to the physiological severity measured by traditional scores.

In contrast, the identification of immobilization situations without the presence of injury, especially in patients discharged early from the emergency room, highlights weaknesses in the decision-making process in PHC and indicates that the decision regarding immobilization has not been fully guided by structured and evidence-based clinical criteria.

Therefore, the results of this study support the need to strengthen continuing education strategies and implement evidence-based care protocols, with an emphasis on careful clinical assessment, recognition of potentially unstable injuries, and decision-making guided by the appropriateness of immobilization to the patient’s clinical condition. From this perspective, selective approaches, such as spinal movement restriction, and interventions with greater scientific support, such as limb immobilization and early pelvic stabilization, should be prioritized.

Thus, improving the quality of care for trauma patients requires the integration of up-to-date scientific knowledge, qualified clinical reasoning, and efficient organization of health services, focusing on tailoring interventions to the presence of injury, contributing to the reduction of unnecessary practices, optimization of resource use, and improvement of clinical outcomes.

Furthermore, considering the scarcity of scientific publications on the subject, the development of national and prospective multicenter studies is recommended to deepen the understanding of the effectiveness of immobilization practices and their implications for the clinical outcomes of trauma patients, especially in the context of PHC.

### Study Limitations

Among the limitations of this study, it is noteworthy that, although the hospital investigated constitutes the main referral unit for trauma care in the state, conducting the research in a single center limits the generalization of the findings to other healthcare settings, especially those with different levels of complexity, network organization, or epidemiological profile. The study sample size should also be considered a limitation. Moreover, the use of data from patient records to identify injuries depended on the quality of the records and the completeness of the information provided by the professionals.

### Contribution to the Field of Nursing, Health, or Public Policy

This study provides relevant contributions to PHC practice, particularly regarding immobilization with injury and its relationship with the clinical progression and prognosis of trauma patients. The findings demonstrate potential applicability in clinical practice by assisting nurses in the early identification of patients with greater care complexity and a higher risk of adverse outcomes, strengthening clinical reasoning, decision-making, and prioritization of care based on more precise clinical criteria aligned with the patient’s needs.

Likewise, by highlighting potential instances of inadequate immobilization, the results reinforce the need to improve healthcare practices, with an emphasis on thorough clinical assessment and the adoption of interventions proportionate to the patient’s condition. On this basis, the study contributes to strengthening evidence-based approaches and reducing potentially unnecessary or iatrogenic practices.

These findings are particularly relevant in settings marked by structural and operational limitations, such as those observed in the Northern Region of Brazil, where the suitability of interventions to the presence of injuries and the optimization of resources are crucial for the quality of care. In the context of health management, the evidence produced can support the planning, organization, and improvement of the Emergency and Urgent Care Network, contributing to the qualification of care practices, the standardization of procedures, and the strengthening of evidence-based strategies in trauma care.

## CONCLUSIONS

The findings of this study show that immobilization with injury in the context of trauma is associated with clinical and care characteristics that reflect greater complexity of care, including longer hospital stays, need for circulatory support in PHC, performance of diagnostic tests, especially those focused on investigating osteoarticular injuries, and referral to more complex units. Furthermore, immobilized patients who presented with injuries had a higher mortality rate.

The high proportion of situations where immobilization was inadequate, evidenced by the use of the technique and the absence of injury, shows weaknesses in decision-making in PHC, possibly related to the indiscriminate application of this intervention. This scenario reinforces the need to reorient care protocols, emphasizing systematic clinical assessment, the use of validated screening tools, and the incorporation of up-to-date scientific evidence.

Thus, the strategic role of nursing and the multidisciplinary team in improving the quality of care for trauma patients is underscored, especially with regard to clinical judgment, decision-making, and assurance that interventions are appropriate to the presence of injury. The implementation of continuous training processes, combined with the strengthening of monitoring and evaluation systems for healthcare practice, can contribute to the reduction of unnecessary procedures, optimization of resources, and improvement of clinical outcomes in trauma victims.

## Data Availability

All the data supporting the results of this study were published in the article itself.
