# Supplementary material for: Immobilization of trauma patients and in-hospital clinical progression
Source: Rev Esc Enferm USP. 2026 Aug 17;60:e20250552. doi: 10.1590/1980-220X-REEUSP-2025-0552en (PMC13480123; doi:10.1590/1980-220X-REEUSP-2025-0552en)
Supplement: Translation

# Translation: Imobilização de pacientes traumatizados e evolução clínica intra-hospitalar

**DOI:** 10.1590/1980-220X-REEUSP-2025-0552pt

**Authors:** Anne de Aguiar Gondim, Keyliane Costa de Souza, Quíria Ribeiro da Silva Monteiro, Lilia de Souza Nogueira, Antônia Regynara Moreira Rodrigues, Greiciane da Silva Rocha

**Affiliations:** 1 Universidade Federal do Acre, Centro de Ciências da Saúde e do Desporto, Rio Branco, AC, Brasil.; 2 Hospital de Urgência e Emergência de Rio Branco, Secretaria de Saúde do Estado do Acre, Rio Branco, AC, Brasil.; 3 Universidade de São Paulo, Escola de Enfermagem, Departamento de Enfermagem Médico-Cirúrgica, São Paulo, SP, Brasil.

**Keywords:** Assistência pré-hospitalar, Traumatologia, Imobilização, Evolução clínica, Enfermagem

## Abstract

**Objetivo:**

Analisar a associação entre a imobilização de pacientes traumatizados, considerando a presença ou ausência de lesão, e a evolução clínica intra-hospitalar.

**Método:**

Estudo de coorte prospectiva que analisou traumatizados com idade ≥18, que receberam atendimento pré-hospitalar e foram admitidos em hospital do Acre, Brasil, entre setembro/dezembro de 2023. As variáveis foram organizadas em dimensões epidemiológicas, clínicas, assistenciais e de desfecho. A comparação entre os grupos (com e sem lesão) foi realizada por análise inferencial, com nível de significância de 5%.

**Resultados:**

Pacientes imobilizados e com lesão apresentaram maior complexidade assistencial com necessidade de intervenções, realização de exames diagnósticos e encaminhamento para unidades especializadas, além de maior tempo de internação e frequência de desfecho desfavorável, como óbito. Evidenciou-se importante inadequação na prática de imobilização, caracterizada pela sua realização em pacientes sem lesão, indicando inconsistências na tomada de decisão clínica.

**Conclusão:**

A imobilização esteve associada à maior complexidade do cuidado, porém sua inadequação reforça a necessidade de reorientação das práticas assistenciais com base em evidências científicas.

## INTRODUÇÃO

As causas externas constituem importante problema de saúde pública em escala global, sendo responsáveis por elevada carga de morbidade, hospitalizações e mortalidade evitável. As causas externas incluem acidentes de trânsito, agressões, quedas, afogamentos e outras lesões intencionais ou não intencionais, que impactam significativamente nos indicadores de morbimortalidade, especialmente entre adultos jovens e populações economicamente ativas^(1,2)^.

No Brasil, a mortalidade por causas externas tem apresentado tendência de crescimento nos últimos anos. Em 2022, a taxa nacional atingiu 61,7 óbitos por 100 mil habitantes, elevando-se para 63,3 em 2023 e alcançando 62,8 em 2024. A situação é ainda mais preocupante na Região Norte, onde os indicadores são mais elevados. No ano de 2024, o estado do Acre registrou taxa de 62,0 óbitos por 100 mil habitantes, enquanto a capital, Rio Branco, apresentou 69,1 óbitos por 100 mil habitantes, evidenciando a magnitude do problema e a necessidade de fortalecimento das estratégias de prevenção, vigilância e organização da rede de atenção às urgências e emergências^(3)^.

Além da mortalidade, as lesões decorrentes de causas externas geram elevada demanda por serviços de saúde. Em 2025, foram registradas 1.094.124 internações por essas causas no Brasil, das quais 118.875 ocorreram na Região Norte. No estado do Acre, contabilizaram-se 4.257 internações, sendo 2.703 em Rio Branco, demonstrando a expressiva demanda hospitalar associada a esses agravos. Nesse contexto, torna-se fundamental garantir a integralidade da assistência ao traumatizado, abrangendo o atendimento pré-hospitalar, o cuidado hospitalar e as ações de reabilitação^(4)^.

A qualidade do atendimento inicial ao paciente traumatizado é determinante para o desfecho clínico. Essa assistência inicia-se ainda na cena do evento traumático, com identificação do mecanismo de trauma, avaliação primária e secundária, estabilização e transporte seguro, conforme preconizado pelos protocolos do *Prehospital Trauma Life Support* (PHTLS) e do *Advanced Trauma Life Support* (ATLS)^(5,6)^. No âmbito da enfermagem, destaca-se o *Advanced Trauma Care for Nurses* (ATCN), programa que capacita enfermeiros para a avaliação sistemática e o manejo de traumatizados, fortalecendo a atuação clínica e a tomada de decisão no cuidado ao paciente^(7)^.

Entre as intervenções frequentemente utilizadas no manejo inicial do traumatizado destacam-se as imobilizações, incluindo a estabilização cervical e a restrição do movimento da coluna por meio de dispositivos como colares cervicais, pranchas rígidas e protetores laterais de cabeça, além de imobilização de extremidades com suspeita de fratura(s) com auxílio de talas^(8)^. A imobilização da coluna previne lesões adicionais e a de extremidades tem como objetivos reduzir a dor e o sangramento, evitar deslocamentos adicionais de fragmentos ósseos e prevenir danos secundários às estruturas adjacentes, contribuindo para maior estabilidade da lesão e transporte seguro do traumatizado^(5)^.

A indicação de imobilização no atendimento ao traumatizado deve estar diretamente relacionada à suspeita ou confirmação de lesão, especialmente na presença de risco de instabilidade da coluna vertebral e/ou comprometimento neurológico. Diretrizes internacionais^(5,6)^ recomendam que essa decisão seja fundamentada em critérios clínicos bem estabelecidos, como o mecanismo do trauma, os achados do exame físico e a presença de rebaixamento do nível de consciência do traumatizado. No entanto, observa-se, na prática assistencial, a realização desnecessária de imobilizações em pacientes com nível de consciência preservado e sem evidência de lesão(ões) ou de mecanismo de trauma importante, o que pode contribuir para o aumento do desconforto e do risco de complicações. Essa condição evidencia fragilidades no processo de tomada de decisão clínica e reforça a necessidade de uma abordagem criteriosa, orientada por evidências, que assegure a segurança do paciente e a adequação das intervenções no contexto do trauma.

Adicionalmente, evidências contemporâneas têm questionado a aplicação universal da imobilização espinhal com dispositivos rígidos. Nesse contexto, diretrizes atuais propõem a abordagem de *Spinal Motion Restriction* (SMR), baseada na restrição seletiva do movimento da coluna a partir de uma avaliação clínica individualizada. O uso indiscriminado de colares cervicais e pranchas rígidas tem sido associado a efeitos adversos relevantes, incluindo dor e desconforto, aumento da pressão sobre tecidos moles, risco de lesões por pressão, elevação da pressão intracraniana e comprometimento da mecânica respiratória^(9,10,11)^.

Neste contexto, o atendimento ao paciente traumatizado é multiprofissional, com participação central da equipe de enfermagem na avaliação clínica, tomada de decisão e implementação de intervenções de estabilização, incluindo procedimentos de imobilização ortopédica, conforme estabelecido pelas Resoluções do Conselho Federal de Enfermagem (COFEN) nº 713/2022 e nº 705/2022^(12,13)^.

Apesar da relevância das imobilizações para a segurança e o prognóstico dos traumatizados, a literatura nacional ainda apresenta lacunas importantes sobre o tema. Observa-se escassez de evidências que permitam compreender, de forma mais robusta, a prática rotineira de imobilização de traumatizados e sua possível relação com presença de lesão e desfechos clínicos. Ademais, observa-se carência de investigações desenvolvidas na Região Amazônica, especialmente no estado do Acre, que abordem essa temática e analisem o papel da enfermagem no manejo inicial e hospitalar de traumatizados. Por conseguinte, o presente estudo teve como objetivo analisar a associação entre a imobilização em pacientes traumatizados, considerando a presença ou ausência de lesão, e a evolução clínica intra-hospitalar.

## MÉTODO

### Aspectos Éticos

Este projeto de pesquisa foi analisado e aprovado pelo Comitê de Ética em Pesquisa da Universidade Federal do Acre sob o parecer nº 5.870.278 e com certificado de apresentação para a apreciação ética (CAAE) nº 64424822.3.0000.5020. A condução da pesquisa atendeu integralmente aos preceitos éticos estabelecidos pelas Resoluções do Conselho Nacional de Saúde, assegurando o respeito aos princípios da dignidade humana, autonomia, beneficência, não maleficência e justiça. Foram garantidos o sigilo e a confidencialidade das informações, bem como a proteção integral dos participantes em todas as etapas do estudo.

### Desenho, Período e Local do Estudo

Trata-se de um estudo de coorte prospectiva, conduzido no período de setembro a dezembro de 2023, no Hospital de Urgência e Emergência de Rio Branco (HUERB), Acre, Brasil. O HUERB é uma unidade hospitalar pública de grande porte, vinculada ao Sistema Único de Saúde (SUS), que atua como referência estadual para o atendimento de urgência e emergência, abrangendo pacientes provenientes de todos os municípios do estado. A instituição dispõe de estrutura assistencial completa (centro cirúrgico, unidade de terapia intensiva, leitos de observação e unidades de internação especializada) para atendimento de pacientes com condições clínicas, cirúrgicas e traumáticas.

### População ou Amostra

A população do estudo foi composta por traumatizados atendidos no setor de emergência traumática do HUERB. A amostra foi por conveniência e considerou os pacientes com idade igual ou superior a 18 anos, que sofreram trauma contuso, penetrante ou misto (contuso e penetrante), receberam atendimento pré-hospitalar (APH) e foram admitidos na emergência do HUERB em até 24 horas do evento traumático. Foram excluídos pacientes readmitidos no HUERB por complicações de lesões traumáticas prévias.

### Protocolo de Estudo

A coleta de dados foi realizada diariamente, no período de setembro a dezembro de 2023, nos turnos diurno e noturno em esquema de plantão dos pesquisadores. As informações foram coletadas por meio de instrumento previamente elaborado pelos pesquisadores que contemplava variáveis clínicas e epidemiológicas relevantes para a comparação entre os grupos de pacientes imobilizados com e sem lesão. Os dados coletados foram inseridos na plataforma REDCap, garantindo a segurança das informações.

As variáveis analisadas foram organizadas em quatro dimensões principais: epidemiológica, clínica, assistencial e de desfecho. A variável independente do estudo foi com presença de lesão(ões) (sim e não) nos pacientes identificada(s) pelos exames de imagens, descrição cirúrgica e registros dos profissionais em prontuário eletrônico. As variáveis da dimensão epidemiológica incluíram sexo e idade dos pacientes, as características do trauma como causa externa (acidente de trânsito, queda, lesão acidental, agressão e lesão autoprovocada), local de ocorrência (zona urbana ou rural) e tipo de trauma (contuso, penetrante ou misto). Em seguida, analisaram-se as intervenções (dimensão assistencial) realizadas nos atendimentos pré e intra-hospitalar, contemplando o tipo de imobilização no APH e de suporte prestado (básico ou avançado), assim como os suportes respiratório e circulatório, tanto no APH quanto no ambiente intra-hospitalar.

Também foram incluídos indicadores da dimensão clínica, compostos por variáveis como tempo de permanência na unidade de emergência e na unidade de terapia intensiva (UTI), de ventilação mecânica e de internação hospitalar, além dos escores de gravidade *Revised Trauma Score* (RTS)^(14)^; no atendimento pré e intra-hospitalar, utilizou-se o *Rapid Emergency Medicine Score* (REMS)^(15)^ e no ambiente intra-hospitalar, o modified REMS (mREMS^(16)^). Adicionalmente, foram analisadas variáveis relacionadas à investigação diagnóstica, avaliações especializadas e complicações clínicas, incluindo a realização de exames de imagem (radiografias de tórax, pelve, membros superiores, membros inferiores e pé, bem como tomografias de coluna cervical, coluna e abdome/pelve), avaliações por especialistas (ortopedista, cirurgião vascular e neurocirurgião) e ocorrência de complicações, como pneumonia associada à ventilação mecânica, lesão por pressão, infecção do sítio cirúrgico e lesão renal aguda.

Por fim, foram consideradas as variáveis da quarta dimensão, incluindo o destino do traumatizado após o atendimento na emergência (alta hospitalar, encaminhamento para unidade de ortopedia, centro cirúrgico, unidade de internação, unidade de terapia intensiva, unidade de observação, evasão ou óbito) e o desfecho final (alta hospitalar, evasão, óbito, transferência para outro hospital, alta a pedido e outros tipos de alta). Esse conjunto de variáveis possibilitou uma análise abrangente da associação entre imobilização com e sem lesão e o perfil do trauma, as intervenções assistenciais, a gravidade clínica, a complexidade do cuidado e os desfechos dos pacientes traumatizados.

Os dados foram coletados durante a permanência do traumatizado na sala de emergência, incluindo manejo clínico, avaliações especializadas, exames realizados, tempo de permanência na unidade e destino após atendimento. Nos casos em que houve internação, as informações complementares foram obtidas prospectivamente no prontuário eletrônico institucional (sistema G-HOSP), contemplando diagnóstico, admissão em UTI, ocorrência de complicações, tempo total de internação e desfecho clínico final.

### Análise dos Recursos e Estatísticas

As variáveis categóricas foram descritas por frequências absoluta (n) e relativa (%) e as variáveis numéricas por medidas de tendência central e dispersão. Para a análise comparativa entre os grupos (com e sem lesão), foram empregados testes estatísticos conforme a natureza das variáveis e a distribuição dos dados. A normalidade das variáveis numéricas foi avaliada por meio do teste de *Shapiro-Wilk* e a homogeneidade das variâncias pelo teste de *Levene*. Diante da violação dos pressupostos de normalidade e/ou homocedasticidade, optou-se pela utilização de testes não paramétricos, incluindo o teste de *Wilcoxon-Mann-Whitney* e o teste de *Brunner-Munzel*, conforme a adequação.

A comparação dos grupos segundo variáveis categóricas foi realizada por meio do teste do qui-quadrado de Pearson ou exato de Fisher. Adotou-se nível de significância de 5% (p < 0,05) para todas as análises. As análises estatísticas foram realizadas no software R (*R Foundation for Statistical Computing, Viena, Áustria*), versão 4.5.2.

## RESULTADOS

Foram registradas 299 admissões hospitalares na unidade de emergência traumática no período de setembro a dezembro de 2023. Destas, 162 receberam APH, constituindo a amostra final do estudo, e 39 (24,1%) apresentaram lesão(ões) identificada(s) em exames de imagens, descrição cirúrgica e registros em prontuários. Na amostra, observou-se predomínio do sexo masculino (68,5%), com média e mediana de idade de 37,5 (DP 14,3) e 34,5 anos, respectivamente, e variação de 19 e 91 anos. Não houve diferença significativa entre os pacientes com e sem lesão em relação ao sexo e idade (p > 0,05).

A distribuição dos pacientes segundo as características do trauma evidenciou que os acidentes de trânsito constituíram a principal causa externa em ambos os grupos, com maior proporção entre os pacientes com presença de lesão (76,9%) em comparação àqueles sem lesão (57,8%). Não houve diferença significativa entre os grupos em relação à causa externa (p = 0,274). Em relação ao local de ocorrência do trauma, observou-se predomínio de eventos em área urbana, tanto entre os pacientes sem lesão (82,1%) quanto entre aqueles com lesão (84,6%), não sendo identificada associação significativa nesta análise comparativa (p = 0,720) (Tabela 1).

**Tabela 1:** Pacientes (n = 162) segundo presença de lesão e características do trauma – Rio Branco, AC, Brasil, 2023.

Variáveis	Presença de lesão		Valor de p
	Não	Sim	
	n (%)	n (%)	
Causa externa			
Acidente de trânsito	71 (57,7)	30 (76,9)	
Queda	19 (15,5)	2 (5,1)	0,274*
Lesão acidental	5 (4,1)	2 (5,1)	
Agressão	26 (21,1)	5 (12,9)	
Lesão autoprovocada	2 (1,6)	-	
Local da ocorrência			
Zona urbana	101 (82,1)	33 (84,6)	0,720**
Zona rural	22 (17,9)	6 (15,4)	
Tipo de trauma			
Contuso	80 (65,1)	18 (46,2)	< 0,001*
Misto	19 (15,4)	19 (48,7)	
Penetrante	24 (19,6)	2 (5,1)	

*Teste exato de Fisher;

**Teste qui-quadrado de Pearson.

Por outro lado, observa-se ainda na Tabela 1 que o tipo de trauma demonstrou associação estatisticamente significativa entre pacientes com lesão (p < 0,001), sendo o trauma contuso mais frequente entre aqueles sem lesão (65,1%) e os traumas mistos (contuso e penetrante) com lesão (48,7%). O trauma penetrante isolado, por sua vez, foi mais frequente no grupo sem lesão (19,6%).

Quanto à dimensão assistencial (Tabela 2), observou-se associação entre os pacientes com e sem lesão e as intervenções assistenciais no APH: imobilização (p < 0,001), suporte circulatório básico (p = 0,014) e suporte circulatório avançado (p = 0,005). Todos os pacientes com lesão foram submetidos à imobilização e, entre aqueles sem lesão, 56,9% também foram imobilizados. Ademais, o suporte circulatório básico realizado no APH foi mais frequente entre os pacientes com lesão (41,0%) em comparação aos sem lesão (21,1%). Da mesma forma, o suporte circulatório avançado, também realizado no contexto pré-hospitalar, apresentou maior frequência entre os pacientes com lesão (89,7%), evidenciando maior necessidade de intervenções hemodinâmicas nesse grupo. Não houve diferença significativa entre os grupos (p > 0,050) em relação ao atendimento e suporte respiratório (básico ou avançado) no pré-hospitalar e aos suportes respiratório e circulatório no intra-hospitalar.

**Tabela 2:** Pacientes (n = 162) segundo presença de lesão e intervenções assistenciais no atendimento pré e intra-hospitalar – Rio Branco, AC, Brasil, 2023.

Variáveis	Presença de lesão		Valor de p
	Não	Sim	
	n (%)	n (%)	
Imobilização no APH			
Não	53 (43,1)	-	<0,001*
Sim	70 (56,9)	39 (100,0)	
Tipo de suporte			
Básico	97 (78,9)	32 (82,1)	0,668**
Avançado	26 (21,1)	7 (17,9)	
Suporte respiratório básico no APH			
Não	112 (91,1)	36 (92,3)	0,809**
Sim	11 (8,9)	3 (7,7)	
Suporte respiratório avançado no APH			
Não	117 (95,1)	37 (94,9)	0,950**
Sim	6 (4,9)	2 (5,1)	
Suporte circulatório básico no APH			
Não	97 (78,9)	23 (59,0)	0,014**
Sim	26 (21,1)	16 (41,0)	
Suporte circulatório avançado no APH			
Não	41 (33,3)	4 (10,3)	0,005**
Sim	82 (66,7)	35 (89,7)	
Suporte respiratório no intra-hospitalar			
Não	109 (88,6)	35 (92,1)	0,542**
Sim	14 (11,4)	3 (7,9)	
Suporte circulatório no intra-hospitalar			
Não	53 (43,1)	17 (43,6)	0,956**
Sim	70 (56,9)	22 (56,4)	

*Teste exato de Fisher;

**Teste qui-quadrado de Pearson.

Legenda: APH – Atendimento Pré-Hospitalar.

Os dados na Tabela 3 mostram que os grupos foram semelhantes em relação à gravidade do trauma mensurada pelos índices RTS no pré-hospitalar (p = 0,480) e RTS (p = 0,404), REMS (p = 0,607) e mREMS (p = 0,839) no intra-hospitalar, assim como o tempo de permanência na unidade de emergência (p = 0,265) e na UTI (p = 0,561) e de uso de ventilação mecânica (p = 0,108). Por outro lado, o tempo de internação hospitalar apresentou diferença estatisticamente significativa entre os grupos (p < 0,001), sendo maior entre os pacientes imobilizados e com lesão (mediana de 3 dias) em comparação àqueles sem lesão (0 dia).

**Tabela 3:** Estatísticas descritivas dos indicadores de dimensão clínica segundo presença de lesão – Rio Branco, AC, Brasil, 2023 (n = 162).

Variáveis	Presença de lesão		p
	Não	Sim	
	Mediana (Q1–Q3)		
Tempo na unidade de emergência (em horas)	2,6 (1,5 – 4,7)	2,0 (1,1 – 4,5)	0,265*
Tempo na UTI (em dias)	14 (6 – 19)	14 (13,2 – 17,2)	0,561*
Tempo de ventilação mecânica (em dias)	4 (3 – 6,5)	11 (9,5 – 12,5)	0,108*
Tempo de internação hospitalar	0 (0 – 1)	3 (2 – 5)	<0,001**
RTS - APH	12 (12)	12 (12)	0,480*
RTS – Intra-hospitalar	7,8 (7,8 – 7,8)	7,8 (7,8 – 7,8)	0,404*
REMS – Intra-hospitalar	2 (0 – 4)	2 (0 – 3)	0,697*
mREMS – Intra-hospitalar	1 (0 – 2)	1 (0,2)	0,839*

*Teste Wilcoxon-Mann-Whitney;

**Teste Brunner-Munzel.

Legenda: UTI – Unidade de Terapia Intensiva; RTS – Revised Trauma Score; REMS – Rapid Emergency Medicine Score; mREMS – modified Rapid Emergency Medicine Score.

A análise da associação entre presença ou não de lesão e as investigações diagnósticas, avaliações especializadas e complicações clínicas evidenciou diferenças significativas em algumas variáveis específicas (Tabela 4). Em relação aos exames de imagem, a radiografia de membros superiores foi mais frequente entre os pacientes imobilizados com lesão (51,3%) em comparação aos sem lesão (13,8%) (p < 0,001). De forma semelhante, a radiografia de membros inferiores também foi significativamente mais realizada no grupo com lesão (69,2%) (p < 0,001), sugerindo maior ocorrência de lesões ortopédicas nesses pacientes. No que se refere às avaliações especializadas, a avaliação do ortopedista (p < 0,001) foi mais frequente entre os pacientes imobilizados com lesão (87,1%) em comparação aos sem lesão (27,6%).

**Tabela 4:** Pacientes (n = 162) segundo presença de lesão e exames diagnósticos, avaliações especializadas e complicações clínicas – Rio Branco, AC, Brasil, 2023.

Variáveis	Presença de lesão		Valor de p
	Não	Sim	
	n (%)	n (%)	
Radiografia de tórax			
Não	53 (43,1)	14 (35,9)	0,428*
Sim	70 (56,9)	25 (64,1)	
Radiografia de pelve			
Não	72 (58,5)	19 (48,7)	0,283*
Sim	51 (41,5)	20 (51,3)	
Radiografia de MMSS			
Não	106 (86,2)	19 (48,7)	<0,001*
Sim	17 (13,8)	20 (51,3)	
Radiografia de MMII			
Não	90 (73,2)	12 (30,8)	<0,001*
Sim	33 (26,8)	27 (69,2)	
Tomografia de cervical			
Não	95 (77,2)	31 (79,5)	0,769*
Sim	28 (22,8)	8 (20,5)	
Tomografia de coluna			
Não	99 (80,5)	33 (84,6)	0,564*
Sim	24 (19,5)	6 (15,4)	
Avaliação do Ortopedista			
Não	89 (72,4)	5 (12,8)	<0,001*
Sim	34 (27,6)	34 (87,1)	
Avaliação do Cirurgião Vascular			
Não	120 (97,6)	39 (100,0)	1,000**
Sim	3 (2,4)	-	
Avaliação do Neurocirurgião			
Não	75 (61,0)	26 (66,7)	0,524*
Sim	48 (39,0)	13 (33,3)	
Internação em UTI			
Não	116 (94,3)	35 (89,7)	0,325*
Sim	7 (5,7)	4 (10,3)	
Pneumonia associada à VM			
Não	122 (99,2)	37 (94,9)	0,144**
Sim	1 (0,8)	2 (5,1)	
Lesão por pressão			
Não	122 (99,2)	37 (94,9)	0,144**
Sim	1 (0,8)	2 (5,1)	
Infecção do sítio cirúrgico			
Não	122 (99,2)	37 (94,9)	0,144**
Sim	1 (0,8)	2 (5,1)	
Lesão renal aguda			
Não	122 (99,2)	37 (94,9)	0,144**
Sim	1 (0,8)	2 (5,1)	

*Teste qui-quadrado de Pearson;

**Teste exato de Fisher.

Legenda: MMSS – Membros superiores; MMII – Membros inferiores; UTI – Unidade de terapia intensiva; VM – Ventilação mecânica.

Quanto às complicações clínicas (pneumonia associada à ventilação mecânica, lesão por pressão, infecção do sítio cirúrgico e lesão renal aguda), não foram identificadas associações estatisticamente significativas entre os grupos (p = 0,144). De modo geral, esses resultados destacam que pacientes imobilizados e que apresentaram lesão apresentaram condições associadas ao sistema musculoesquelético, como maior realização de exames radiográficos de membros e avaliação ortopédica (Tabela 4).

Os dados da Tabela 5 mostram maior frequência de alta hospitalar após o atendimento na emergência entre os pacientes sem lesão (68,4%) em comparação aos com lesão (10,3%). Por outro lado, os pacientes imobilizados com lesão apresentaram maior encaminhamento para unidades de maior complexidade assistencial, destacando-se a unidade de ortopedia (33,3%), o centro cirúrgico (23,1%) e unidades de internação (23,1%), evidenciando diferenças entre os grupos (p < 0,001).

**Tabela 5:** Pacientes (n = 162) segundo presença de lesão e destino após atendimento na emergência e desfecho final – Rio Branco, AC, Brasil, 2023.

Variáveis	Presença de lesão		p*
	Não	Sim	
	n (%)	n (%)	
Destino após emergência			
Alta hospitalar	84 (68,4)	4 (10,3)	
Unidade de ortopedia	11 (8,9)	13 (33,3)	<0,001
Centro cirúrgico	12 (9,8)	9 (23,1)	
Unidade internação	9 (7,3)	9 (23,1)	
Unidade de terapia intensiva	4 (3,2)	3 (7,7)	
Evasão	1 (0,8)	-	
Unidade observação	1 (0,8)	-	
Óbito	-	1 (2,5)	
Outro	1 (0,8)	-	
Desfecho final			
Alta hospitalar	109 (94,8)	32 (84,2)	
Evasão	3 (2,6)	1 (2,6)	
Óbito	2 (1,7)	3 (7,9)	0,044
Outros tipos de alta	1 (0,9)	-	
Transferência para outro hospital	-	1 (2,6)	
Alta a pedido	-	1 (2,6)	

*Teste exato de Fisher.

No que se refere ao desfecho da internação (Tabela 5), também foi observada associação estatisticamente significativa (p = 0,044) entre os grupos analisados no estudo. A alta hospitalar foi mais frequente entre os pacientes sem lesão (94,8%) e a ocorrência de óbito entre aqueles com lesão (7,9%). De forma geral, esses achados indicam que traumatizados imobilizados e com lesão apresentam elevada complexidade assistencial e gravidade, evidenciadas por encaminhamento para setores especializados após o atendimento na emergência e maior ocorrência de desfecho desfavorável, como o óbito.

## DISCUSSÃO

Os resultados deste estudo evidenciam que a imobilização com presença de lesão está diretamente relacionada às características do trauma, ao perfil assistencial no atendimento pré- hospitalar e à evolução clínica dos pacientes. No que se refere ao mecanismo do trauma, observa-se que eventos de maior energia, como os acidentes de trânsito, estiveram mais frequentemente associados à imobilização com lesão, corroborando o achado de um estudo^(17)^ que destaca esses eventos como principais responsáveis por lesões graves e potencialmente incapacitantes. Além disso, a maior frequência de indicação em traumas com componentes mistos (contuso/penetrante) reforça que a complexidade do mecanismo lesional influencia diretamente a decisão clínica, conforme discutido nas diretrizes do *American College of Surgeons* ^(18)^, que enfatizam o mecanismo do trauma como um dos pilares da avaliação inicial.

No contexto do APH, os resultados demonstram coerência parcial (mais de 50% dos pacientes do estudo foram imobilizados e não apresentavam lesão) entre a avaliação clínica e a realização da imobilização, indicando que essa intervenção permanece fortemente incorporada à prática assistencial. A ocorrência de imobilizações em pacientes sem indicação clínica clara evidencia a persistência de um modelo pautado na precaução universal. Esse achado é consistente com a literatura contemporânea^(18,19)^, que aponta a imobilização frequentemente aplicada de forma ampliada, mesmo na ausência de evidências robustas que sustentem seu uso indiscriminado. Diretrizes recentes têm recomendado a transição para estratégias baseadas no conceito de *Spinal Motion Restriction* (SMR), que orienta a restrição seletiva do movimento da coluna a partir de critérios clínicos bem definidos^(9)^.

Nesse sentido, quando não é possível excluir com segurança a presença de lesão, especialmente em cenários de avaliação limitada no APH, rebaixamento de consciência e intoxicação, a restrição do movimento pode ser indicada como medida de precaução. Contudo, deve-se evitar o uso indiscriminado de dispositivos rígidos, como a prancha longa, priorizando abordagens mais seguras, confortáveis e individualizadas. Assim, a prática atual não respalda a imobilização rotineira diante da incerteza, mas sim uma tomada de decisão clínica estruturada, que equilibre riscos e benefícios para o paciente^(9)^.

Adicionalmente, a associação observada entre a presença de lesão e a maior necessidade de suporte circulatório no atendimento pré-hospitalar sugere que esses pacientes apresentam maior comprometimento fisiológico e hemodinâmico. Esse achado reforça a relação entre a indicação de imobilização e a gravidade clínica do traumatizado, mostrando que a intervenção tem sido empregada com maior frequência em cenários de maior complexidade assistencial. Tal resultado está em consonância com a literatura, que demonstra que intervenções mais avançadas no contexto pré-hospitalar tendem a concentrar-se em pacientes com maior severidade do trauma e maior risco de instabilidade clínica^(20)^.

Assim, a imobilização nesse contexto pode ser interpretada como um marcador indireto de severidade, refletindo mais a percepção clínica de risco pelas equipes assistenciais do que uma intervenção isoladamente determinante de desfechos. Essa interpretação reforça que sua indicação tende a ocorrer em pacientes com maior comprometimento fisiológico e potencial de instabilidade. Em consonância com esses achados, a literatura aponta que, no cenário pré-hospitalar, a equipe de enfermagem desempenha papel central na estabilização hemodinâmica e clínica do paciente traumatizado, atuando no suporte circulatório e respiratório por meio de intervenções como punção venosa, administração de medicamentos, monitoramento contínuo de sinais vitais, oferta de oxigenoterapia e realização de curativos^(21)^. Nesse sentido, a imobilização deve ser compreendida como parte de um conjunto integrado de cuidados, que inclui também a manutenção da perfusão tecidual e a prevenção de agravos secundários.

No que se refere à evolução clínica, verificou-se que pacientes imobilizados com lesão apresentaram maior tempo de internação hospitalar, o que reforça sua associação com maior complexidade assistencial. Embora os escores de gravidade não tenham demonstrado diferenças expressivas entre os grupos, esse achado sugere que a avaliação clínica inicial e o julgamento profissional podem identificar dimensões da gravidade não completamente mensuradas por escalas fisiológicas. Esse fenômeno é discutido por pesquisadores^(22)^ ao destacarem que a tomada de decisão no trauma é multifatorial, envolvendo não apenas parâmetros objetivos, mas também o mecanismo do trauma, o contexto do atendimento e a experiência do profissional.

No ambiente intra-hospitalar, os pacientes imobilizados com lesão foram mais frequentemente submetidos a exames diagnósticos e avaliações especializadas, especialmente na área ortopédica. Esse resultado reforça a associação entre adequação de imobilização e suspeita de lesões estruturais^(23)^. A maior utilização de exames de imagem nesses pacientes pode refletir tanto a maior gravidade quanto a necessidade de confirmação diagnóstica diante da possibilidade de lesões ocultas, evidenciando uma abordagem mais cautelosa por parte das equipes assistenciais.

No que se refere ao destino após atendimento na emergência e aos desfechos hospitalares, observou-se que pacientes imobilizados e com lesão apresentaram maior necessidade de internação em unidades especializadas, maior frequência de intervenções cirúrgicas e maior ocorrência de óbito. Esses achados reforçam que a imobilização está fortemente associada a pacientes com maior gravidade e complexidade clínica^(18,24)^. Entretanto, é fundamental destacar que essa associação não deve ser interpretada como relação causal. Evidências recentes^(25,26)^ indicam que a imobilização pré-hospitalar não está consistentemente associada à redução de déficits neurológicos ou à melhoria dos desfechos clínicos. Dessa forma, a imobilização deve ser compreendida como parte de um conjunto de intervenções direcionadas a pacientes mais graves, e não como fator isolado determinante da evolução clínica.

Diante desse cenário, torna-se evidente a necessidade de reavaliação das práticas assistenciais relacionadas à imobilização no trauma. A literatura aponta para a transição de um modelo baseado na imobilização universal para uma abordagem mais seletiva, fundamentada no conceito de *Spinal Motion Restriction (SMR).* Conforme destacado em estudos e diretrizes^(9,27)^, essa abordagem permite reduzir intervenções desnecessárias, minimizar riscos associados e manter a segurança do paciente, promovendo maior racionalidade no cuidado.

Assim, os resultados deste estudo não apenas reforçam a associação entre imobilização e maior gravidade clínica, mas também evidenciam lacunas na adequação de sua indicação, destacando a importância da incorporação de protocolos clássicos baseados em evidências, como os critérios do *National Emergency X-Radiography Utilization Study* (*NEXUS*) e (*Canadian C-Spine Rule*)^(28,29)^. A adoção dessas estratégias, aliada à capacitação contínua das equipes de saúde, especialmente da enfermagem, que ocupa posição estratégica na avaliação e execução dessas intervenções, pode contribuir para uma assistência mais segura, eficiente e alinhada às melhores evidências científicas disponíveis.

Adicionalmente, quanto às imobilizações de membros e à estabilização da pelve, observa-se que essas intervenções têm apresentado maior respaldo na literatura recente quando comparadas à imobilização rotineira da coluna. Estudos indicam que a imobilização de extremidades, especialmente em casos de fraturas suspeitas ou confirmadas, contribui para a redução da dor, prevenção de lesões adicionais em tecidos moles e diminuição do risco de complicações durante o transporte do paciente^(30)^. No mesmo sentido, a estabilização pélvica precoce, por meio do uso de cintas pélvicas, tem sido recomendada em pacientes com suspeita de fratura instável de pelve, estando associada à redução do volume pélvico, controle do sangramento e melhora da estabilidade hemodinâmica^(30)^.

Diretrizes da *World Society of Emergency Surgery* reforçam que a aplicação precoce de dispositivos de estabilização pélvica no atendimento pré-hospitalar e na sala de emergência constitui medida importante no manejo inicial do trauma pélvico grave^(30)^. Assim, diferentemente da imobilização cervical indiscriminada, as imobilizações de membros e a estabilização pélvica apresentam indicações mais bem estabelecidas e sustentadas por evidências, devendo ser incorporadas de forma criteriosa e precoce nos protocolos assistenciais, contribuindo para a redução de complicações e para a melhoria dos desfechos clínicos.

Em síntese, os achados deste estudo demonstram que a imobilização com lesão está associada a um perfil de pacientes com maior complexidade assistencial no contexto do trauma, evidenciado pela maior frequência em traumas combinados, maior necessidade de intervenções circulatórias no atendimento pré-hospitalar e de realização de exames diagnósticos direcionados ao sistema musculoesquelético, maior demanda por avaliação ortopédica e encaminhamento para unidades de maior densidade tecnológica, como centro cirúrgico e internação hospitalar. Adicionalmente, esses pacientes apresentaram maior tempo de internação e frequência de óbito hospitalar, indicando que a adequação da imobilização se relaciona mais à carga de lesões e à complexidade do cuidado do que à gravidade fisiológica mensurada por escores tradicionais.

Por outro lado, a identificação de situações de imobilização sem presença de lesão, especialmente em pacientes com alta precoce da emergência, evidencia fragilidades no processo decisório no APH e indica que a tomada de decisão quanto à imobilização não tem sido plenamente orientada por critérios clínicos estruturados e baseados em evidências.

Diante disso, os resultados deste estudo sustentam a necessidade de fortalecimento de estratégias de educação permanente e de implementação de protocolos assistenciais baseados em evidências, com ênfase na avaliação clínica criteriosa, no reconhecimento de lesões potencialmente instáveis e na tomada de decisão orientada pela adequação da imobilização à condição clínica do paciente. Nesse contexto, abordagens seletivas, como a restrição de movimento da coluna, e intervenções com maior respaldo científico, como a imobilização de extremidades e a estabilização pélvica precoce, devem ser priorizadas.

Assim, a qualificação da assistência ao paciente traumatizado requer a integração entre conhecimento científico atualizado, raciocínio clínico qualificado e organização eficiente dos serviços de saúde, com foco na adequação das intervenções à presença de lesão, contribuindo para a redução de práticas desnecessárias, otimização do uso de recursos e melhoria dos desfechos clínicos.

Ademais, considerando a escassez de produções científicas sobre a temática, recomenda-se o desenvolvimento de estudos multicêntricos nacionais e prospectivos que permitam aprofundar a compreensão acerca da efetividade das práticas de imobilização e de suas implicações nos desfechos clínicos de pacientes traumatizados, especialmente no âmbito do APH.

### Limitações do Estudo

Entre as limitações deste estudo, destaca-se que, embora o hospital investigado constitua a principal unidade de referência para atendimento ao trauma no estado, a condução da pesquisa em um único centro limita a generalização dos achados para outros contextos assistenciais, especialmente aqueles com diferentes níveis de complexidade, organização da rede ou perfil epidemiológico. O tamanho da amostra do estudo também deve ser considerado como limitação. Adicionalmente, a utilização de dados provenientes de prontuários dos pacientes para identificação das lesões dependeu da qualidade dos registros e completude das informações dos profissionais.

### Contribuição para Área da Enfermagem, Saúde ou Política Pública

Este estudo oferece contribuições relevantes para a prática assistencial no APH, particularmente no que se refere à imobilização com lesão e sua relação com a evolução clínica e o prognóstico de traumatizados. Os achados evidenciam potencial aplicabilidade na prática clínica ao subsidiar o enfermeiro na identificação precoce de pacientes com maior complexidade assistencial e maior risco de desfechos desfavoráveis, fortalecendo o raciocínio clínico, a tomada de decisão e a priorização do cuidado com base em critérios clínicos mais precisos e alinhados às necessidades do paciente.

Adicionalmente, ao evidenciar possíveis situações de inadequação da imobilização, os resultados reforçam a necessidade de qualificação das práticas assistenciais, com ênfase na avaliação clínica criteriosa e na adoção de intervenções proporcionais à condição do paciente. Nesse sentido, o estudo contribui para o fortalecimento de abordagens baseadas em evidências e para a redução de condutas potencialmente desnecessárias ou iatrogênicas.

Os achados assumem especial relevância em cenários marcados por limitações estruturais e operacionais, como aqueles observados na Região Norte do Brasil, onde a adequação das intervenções à presença de lesão e a otimização de recursos são determinantes para a qualidade do cuidado. No âmbito da gestão em saúde, as evidências produzidas podem subsidiar o planejamento, a organização e o aprimoramento da Rede de Atenção às Urgências e Emergências, contribuindo para a qualificação das práticas assistenciais, a padronização de condutas e o fortalecimento de estratégias baseadas em evidências no cuidado ao traumatizado.

## CONCLUSÃO

Os achados deste estudo evidenciam que a imobilização com lesão no contexto do trauma está associada às características clínicas e assistenciais que refletem maior complexidade do cuidado, incluindo maior tempo de internação, necessidade de suporte circulatório no APH, realização de exames diagnósticos, especialmente voltados à investigação de lesões osteoarticulares, e encaminhamento para unidades de maior complexidade. Ademais, pacientes imobilizados e que apresentaram lesão apresentaram maior frequência de óbito.

A elevada proporção de situações de inadequação da imobilização evidenciada pelo uso da técnica e ausência de lesão evidencia fragilidades na tomada de decisão no APH, possivelmente relacionadas à aplicação não criteriosa dessa intervenção. Esse cenário reforça a necessidade de reorientação dos protocolos assistenciais, com ênfase na avaliação clínica sistematizada, na utilização de ferramentas validadas de triagem e na incorporação de evidências científicas atualizadas.

Dessa forma, destaca-se o papel estratégico da enfermagem e da equipe multiprofissional na qualificação do cuidado ao traumatizado, especialmente no que se refere ao julgamento clínico, à tomada de decisão e à garantia da adequação das intervenções à presença de lesão. A implementação de processos formativos contínuos, associada ao fortalecimento de sistemas de monitoramento e avaliação da prática assistencial, pode contribuir para a redução de condutas desnecessárias, otimização de recursos e melhoria dos desfechos clínicos em vítimas de trauma.

## Data Availability

Todo o conjunto de dados que dá suporte aos resultados deste estudo foi publicado no próprio artigo.

# Translation: Inmovilización de pacientes traumatizados y evolución clínica intrahospitalaria

**DOI:** 10.1590/1980-220X-REEUSP-2025-0552es

**Authors:** Anne de Aguiar Gondim, Keyliane Costa de Souza, Quíria Ribeiro da Silva Monteiro, Lilia de Souza Nogueira, Antônia Regynara Moreira Rodrigues, Greiciane da Silva Rocha

**Affiliations:** 1 Universidade Federal do Acre, Centro de Ciências da Saúde e do Desporto, Rio Branco, AC, Brasil.; 2 Hospital de Urgência e Emergência de Rio Branco, Secretaria de Saúde do Estado do Acre, Rio Branco, AC, Brasil.; 3 Universidade de São Paulo, Escola de Enfermagem, Departamento de Enfermagem Médico-Cirúrgica, São Paulo, SP, Brasil.

**Keywords:** Atención Prehospitalaria, Traumatología, Inmovilización, Evolución Clínica, Enfermería

## Abstract

**Objetivo:**

Analizar la asociación entre la inmovilización de pacientes traumatizados, considerando la presencia o ausencia de lesión, y la evolución clínica intrahospitalaria.

**Método:**

Estudio de cohorte prospectivo que analizó a pacientes traumatizados ≥18 años que recibieron atención prehospitalaria y fueron ingresados en un hospital de Acre, Brasil, entre septiembre y diciembre de 2023. Las variables se organizaron en dimensiones epidemiológicas, clínicas, de la atención y de resultados. La comparación entre los grupos (con y sin lesión) se realizó mediante análisis inferencial, con un nivel de significancia del 5%.

**Resultados:**

Los pacientes inmovilizados con lesiones presentaban una mayor complejidad asistencial, requiriendo intervenciones, pruebas diagnósticas y derivaciones a unidades especializadas, además de estancias hospitalarias más prolongadas y mayor frecuencia de resultados desfavorables, como la muerte. Se detectaron deficiencias significativas en las prácticas de inmovilización, caracterizadas por su aplicación a pacientes sin lesiones, lo que indica inconsistencias en la toma de decisiones clínicas.

**Conclusión:**

La inmovilización se ha asociado con una mayor complejidad en la atención, pero su inadecuación refuerza la necesidad de reorientar las prácticas asistenciales basándose en la evidencia científica.

## INTRODUCCIÓN

Las causas externas constituyen un importante problema de salud pública a escala global, siendo responsables de una elevada carga de morbilidad, hospitalizaciones y mortalidad evitable. Las causas externas incluyen accidentes de tránsito, agresiones, caídas, ahogamientos y otras lesiones intencionales o no intencionales, que impactan significativamente en los indicadores de morbilidad y mortalidad, especialmente entre adultos jóvenes y poblaciones económicamente activas^(1,2)^.

En Brasil, la mortalidad por causas externas ha presentado una tendencia al alza en los últimos años. En 2022, la tasa nacional alcanzó los 61,7 decesos por cada 100 mil habitantes, elevándose a 63,3 en 2023 y alcanzando los 62,8 en 2024. La situación es aún más preocupante en la Región Norte, donde los indicadores son más elevados. En el año 2024, el estado de Acre registró una tasa de 62,0 muertes por cada 100 mil habitantes, mientras que la capital, Río Branco, presentó 69,1 muertes por cada 100 mil habitantes, lo que pone de manifiesto la magnitud del problema y la necesidad de fortalecer las estrategias de prevención, vigilancia y organización de la red de atención a urgencias y emergencias^(3)^.

Además de la mortalidad, las lesiones por causas externas generan una elevada demanda de servicios de salud. En 2025, se registraron 1 094 124 hospitalizaciones por estas causas en Brasil, de las cuales 118 875 ocurrieron en la Región Norte. En el estado de Acre, se contabilizaron 4.257 hospitalizaciones, de las cuales 2.703 fueron en Río Branco, lo que demuestra la significativa demanda hospitalaria asociada a estas lesiones. En este contexto, resulta fundamental garantizar la integralidad de la atención al paciente traumatizado, abarcando la atención prehospitalaria, la atención hospitalaria y las acciones de rehabilitación^(4)^.

La calidad de la atención inicial al paciente traumatizado es determinante para el resultado clínico. Esta atención comienza ya en el lugar del evento traumático, con la identificación del mecanismo del trauma, la evaluación primaria y secundaria, la estabilización y el transporte seguro, según lo recomendado por los protocolos del *Prehospital Trauma Life Support* (PHTLS) y del *Advanced Trauma Life Support* (ATLS)^(5,6)^. En el ámbito de la enfermería, destaca el *Advanced Trauma Care for Nurses* (ATCN), un programa que capacita a los enfermeros para la evaluación sistemática y el manejo de pacientes traumatizados, fortaleciendo la actuación clínica y la toma de decisiones en la atención al paciente^(7)^.

Entre las intervenciones que se utilizan con frecuencia en el manejo inicial del traumatizado se destacan las inmovilizaciones, incluyendo la estabilización cervical y la restricción del movimiento de la columna mediante dispositivos como collares cervicales, tablas rígidas y protectores laterales de cabeza, además de la inmovilización de extremidades con sospecha de fracturas con la ayuda de férulas^(8)^. La inmovilización de la columna previene lesiones adicionales y la de las extremidades tiene como objetivos reducir el dolor y el sangrado, evitar desplazamientos adicionales de fragmentos óseos y prevenir daños secundarios a las estructuras adyacentes, contribuyendo a una mayor estabilidad de la lesión y al transporte seguro del traumatizado^(5)^.

La indicación de inmovilización en la atención al traumatizado debe estar directamente relacionada con la sospecha o confirmación de la lesión, especialmente ante el riesgo de inestabilidad de la columna vertebral y/o compromiso neurológico. Las directrices internacionales^(5,6)^ recomiendan que esta decisión se base en criterios clínicos bien establecidos, como el mecanismo del traumatismo, los hallazgos del examen físico y la presencia de disminución del nivel de conciencia del paciente traumatizado. Sin embargo, en la práctica asistencial se observa la realización innecesaria de inmovilizaciones en pacientes con nivel de conciencia preservado y sin evidencia de lesión(es) o de un mecanismo de trauma importante, lo que puede contribuir al aumento del malestar y del riesgo de complicaciones. Esta situación pone de manifiesto las debilidades en el proceso de toma de decisiones clínicas y refuerza la necesidad de un enfoque criterioso, basado en la evidencia, que garantice la seguridad del paciente y la adecuación de las intervenciones en el contexto del traumatismo.

Además, la evidencia contemporánea ha cuestionado la aplicación universal de la inmovilización espinal con dispositivos rígidos. En este contexto, las directrices actuales proponen el enfoque de *Spinal Motion Restriction* (SMR), basado en la restricción selectiva del movimiento de la columna a partir de una evaluación clínica individualizada. El uso indiscriminado de collares cervicales y tablas rígidas se ha asociado con efectos adversos relevantes, incluyendo dolor y malestar, aumento de la presión sobre los tejidos blandos, riesgo de lesiones por presión, elevación de la presión intracraneal y compromiso de la mecánica respiratoria^(9,10,11)^.

En este contexto, la atención al paciente traumatizado es multiprofesional, con una participación central del equipo de enfermería en la evaluación clínica, la toma de decisiones y la implementación de intervenciones de estabilización, incluyendo procedimientos de inmovilización ortopédica, según lo establecido por las Resoluciones del Consejo Federal de Enfermería (COFEN) n.º 713/2022 y n.º 705/2022^(12,13)^.

A pesar de la relevancia de las inmovilizaciones para la seguridad y el pronóstico de los pacientes traumatizados, la literatura nacional aún presenta lagunas importantes sobre el tema. Se observa una escasez de evidencia que permita comprender, de manera más sólida, la práctica rutinaria de inmovilización de pacientes traumatizados y su posible relación con la presencia de lesiones y los resultados clínicos. Además, se observa una falta de investigaciones desarrolladas en la Región Amazónica, especialmente en el estado de Acre, que aborden esta temática y analicen el papel de la enfermería en el manejo inicial y hospitalario de los pacientes traumatizados. Por consiguiente, el presente estudio tuvo como objetivo analizar la asociación entre la inmovilización en pacientes traumatizados, considerando la presencia o ausencia de lesiones, y la evolución clínica intra-hospitalaria.

## MÉTODO

### Aspectos Éticos

Este proyecto de investigación fue analizado y aprobado por el Comité de Ética en Investigación de la Universidad Federal de Acre bajo el dictamen n.º 5.870.278 y con certificado de presentación para la evaluación ética (CAAE) n.º 64424822.3.0000.5020. La realización de la investigación cumplió íntegramente con los preceptos éticos establecidos por las Resoluciones del Consejo Nacional de Salud, garantizando el respeto a los principios de dignidad humana, autonomía, beneficencia, no maleficencia y justicia. Se garantizó la privacidad y la confidencialidad de la información, así como la protección integral de los participantes en todas las etapas del estudio.

### Diseño, Período y Lugar del Estudio

Se trata de un estudio de cohorte prospectivo, realizado entre septiembre y diciembre de 2023, en el Hospital de Urgencias y Emergencias de Rio Branco (HUERB), Acre, Brasil. El HUERB es un gran centro hospitalario público, vinculado al Sistema Único de Salud (SUS), que actúa como centro de referencia estatal para la atención de urgencias y emergencias, atendiendo a pacientes procedentes de todos los municipios del estado. La institución cuenta con una estructura asistencial completa (centro quirúrgico, unidad de terapia intensiva, camas de observación y unidades de hospitalización especializada) para la atención de pacientes con afecciones clínicas, quirúrgicas y traumáticas.

### Población o Muestra

La población del estudio estuvo compuesta por pacientes traumatizados atendidos en el sector de emergencias traumáticas del HUERB. La muestra fue de conveniencia y consideró a los pacientes de 18 años o más que sufrieron un traumatismo contuso, penetrante o mixto (contuso y penetrante), recibieron atención prehospitalaria (APH) y fueron admitidos en la sala de emergencias del HUERB en un plazo de 24 horas desde el evento traumático. Se excluyó a los pacientes readmitidos en el HUERB por complicaciones de lesiones traumáticas previas.

### Protocolo del Estudio

La recolección de datos se realizó diariamente, en el período comprendido entre septiembre y diciembre de 2023, en los turnos diurnos y nocturnos, según el esquema de guardias de los investigadores. La información se recopiló mediante un instrumento elaborado previamente por los investigadores que contemplaba variables clínicas y epidemiológicas relevantes para la comparación entre los grupos de pacientes inmovilizados con y sin lesión. Los datos recopilados se ingresaron en la plataforma REDCap, garantizando la seguridad de la información.

Las variables analizadas se organizaron en cuatro dimensiones principales: epidemiológica, clínica, asistencial y de resultado. La variable independiente del estudio fue la presencia de lesión(es) (sí y no) en los pacientes, identificada(s) mediante exámenes de imágenes, descripción quirúrgica y registros de los profesionales en el expediente médico electrónico. Las variables de la dimensión epidemiológica incluyeron el sexo y la edad de los pacientes, las características del trauma como causa externa (accidente de tránsito, caída, lesión accidental, agresión y lesión autoprovocada), el lugar de ocurrencia (zona urbana o rural) y el tipo de trauma (contuso, penetrante o mixto). A continuación, se analizaron las intervenciones (dimensión asistencial) realizadas en la atención prehospitalaria e intrahospitalaria, incluyendo el tipo de inmovilización en el servicio de urgencias y el tipo de soporte prestado (básico o avanzado), así como los soportes respiratorio y circulatorio, tanto en el servicio de urgencias como en el entorno intrahospitalario.

También se incluyeron indicadores de la dimensión clínica, compuestos por variables como el tiempo de permanencia en la unidad de emergencias y en la unidad de terapia intensiva (UTI), de ventilación mecánica y de hospitalización, además de las puntuaciones de gravedad *Revised Trauma Score* (RTS)^(14)^;en la atención prehospitalaria e intrahospitalaria, se utilizó el *Rapid Emergency Medicine Score* (REMS)^(15)^ y en el entorno intrahospitalario, el REMS modificado (mREMS^(16)^). Además, se analizaron variables relacionadas con la investigación diagnóstica, las evaluaciones especializadas y las complicaciones clínicas, incluyendo la realización de exámenes de imagen (radiografías de tórax, pelvis, miembros superiores, miembros inferiores y pie, así como tomografías de columna cervical, columna y abdomen/pelvis), evaluaciones por especialistas (ortopedista, cirujano vascular y neurocirujano) y la aparición de complicaciones, como neumonía asociada a la ventilación mecánica, úlceras por presión, infección del sitio quirúrgico y lesión renal aguda.

Por último, se consideraron las variables de la cuarta dimensión, incluyendo el destino del paciente traumatizado tras la atención en la sala de emergencias (alta hospitalaria, derivación a la unidad de ortopedia, quirófano, unidad de hospitalización, unidad de terapia intensiva, unidad de observación, fuga o fallecimiento) y el desenlace final (alta hospitalaria, evasión, fallecimiento, traslado a otro hospital, alta a petición y otros tipos de alta). Este conjunto de variables permitió un análisis exhaustivo de la asociación entre la inmovilización con y sin lesión y el perfil del traumatismo, las intervenciones asistenciales, la gravedad clínica, la complejidad de la atención y los resultados de los pacientes traumatizados.

Los datos se recopilaron durante la estancia del paciente traumatizado en la sala de emergencias, incluyendo el manejo clínico, las evaluaciones especializadas, los exámenes realizados, el tiempo de permanencia en la unidad y el destino tras la atención. En los casos en que hubo hospitalización, la información complementaria se obtuvo prospectivamente del expediente médico electrónico institucional (sistema G-HOSP), incluyendo el diagnóstico, el ingreso en la UTI, la aparición de complicaciones, el tiempo total de hospitalización y el resultado clínico final.

### Análisis de los Datos y Estadísticas

Las variables categóricas se describieron mediante frecuencias absolutas (n) y relativas (%), y las variables numéricas mediante medidas de tendencia central y dispersión. Para el análisis comparativo entre los grupos (con y sin lesión), se emplearon pruebas estadísticas de acuerdo con la naturaleza de las variables y la distribución de los datos. La normalidad de las variables numéricas se evaluó mediante la prueba de *Shapiro-Wilk* y la homogeneidad de las varianzas mediante la prueba de *Levene*. Ante el incumplimiento de los supuestos de normalidad y/o homocedasticidad, se optó por el uso de pruebas no paramétricas, incluyendo la prueba de *Wilcoxon-Mann-Whitney* y la prueba de *Brunner-Munzel*, según la idoneidad.

La comparación de los grupos según variables categóricas se realizó mediante la prueba de Chi-cuadrado de Pearson o la prueba exacta de Fisher. Se adoptó un nivel de significación del 5 % (p < 0,05) para todos los análisis. Los análisis estadísticos se realizaron con el software R (*R Foundation for Statistical Computing, Viena, Austria*), versión 4.5.2.

## RESULTADOS

Se registraron 299 ingresos hospitalarios en la unidad de emergencias traumatológicas en el período comprendido entre septiembre y diciembre de 2023. De estos, 162 recibieron atención prehospitalaria, lo que constituyó la muestra final del estudio, y 39 (24,1 %) presentaron lesiones identificadas en exámenes de imagen, descripciones quirúrgicas y registros en historias clínicas. En la muestra se observó un predominio del sexo masculino (68,5 %), con una media y mediana de edad de 37,5 (DE 14,3) y 34,5 años, respectivamente, y una variación de 19 a 91 años. No hubo diferencias significativas entre los pacientes con y sin lesión en cuanto al sexo y la edad (p > 0,05).

La distribución de los pacientes según las características del traumatismo evidenció que los accidentes de tránsito constituyeron la principal causa externa en ambos grupos, con una mayor proporción entre los pacientes con presencia de lesión (76,9 %)en comparación con aquellos sin lesión (57,8 %). No hubo diferencias significativas entre los grupos en relación con la causa externa (p = 0,274). En cuanto al lugar donde ocurrió el trauma, se observó un predominio de eventos en áreas urbanas, tanto entre los pacientes sin lesiones (82,1 %) como entre aquellos con lesiones (84,6 %), sin que se identificara una asociación significativa en este análisis comparativo (p = 0,720) (Tabla 1).

**Tabla 1:** Pacientes (n = 162) según la presencia de lesiones y las características del trauma – Rio Branco, AC, Brasil, 2023.

Variables	Presencia de lesión		Valor de p
	No	Sí	
	n (%)	n (%)	
Causa externa			
Accidente de tráfico	71 (57,7)	30 (76,9)	
Caída	19 (15,5)	2 (5,1)	0,274*
Lesión accidental	5 (4,1)	2 (5,1)	
Agresión	26 (21,1)	5 (12,9)	
Lesión autoinfligida	2 (1,6)	-	
Lugar del suceso			
Zona urbana	101 (82,1)	33 (84,6)	0,720**
Zona rural	22 (17,9)	6 (15,4)	
Tipo de traumatismo			
Contuso	80 (65,1)	18 (46,2)	< 0,001*
Misto	19 (15,4)	19 (48,7)	
Penetrante	24 (19,6)	2 (5,1)	

*Prueba exacta de Fisher;

**Prueba de chi-cuadrado de Pearson.

Por otro lado, también se observa en la Tabla 1 que el tipo de traumatismo mostró una asociación estadísticamente significativa entre los pacientes con lesión (p < 0,001), siendo el traumatismo contuso más frecuente entre aquellos sin lesión (65,1 %) y los traumatismos mixtos (contuso y penetrante) con lesión (48,7 %). El traumatismo penetrante aislado, por su parte, fue más frecuente en el grupo sin lesión (19,6 %).

En cuanto a la dimensión asistencial (Tabla 2), se observó una asociación entre los pacientes con y sin lesiones y las intervenciones asistenciales en el servicio de urgencias: inmovilización (p < 0,001), soporte circulatorio básico (p = 0,014) y soporte circulatorio avanzado (p = 0,005). Todos los pacientes con lesión fueron sometidos a inmovilización y, entre los que no presentaban lesión, el 56,9 % también fue inmovilizado. Además, el soporte circulatorio básico realizado en el servicio de urgencias fue más frecuente entre los pacientes con lesión (41,0 %) en comparación con los que no presentaban lesión (21,1 %). Del mismo modo, el soporte circulatorio avanzado, también realizado en el contexto prehospitalario, se observó con mayor frecuencia entre los pacientes con lesión (89,7 %), lo que pone de manifiesto una mayor necesidad de intervenciones hemodinámicas en este grupo. No hubo diferencias significativas entre los grupos (p > 0,050) en relación con la atención y el soporte respiratorio (básico o avanzado) en el ámbito prehospitalario y con los soportes respiratorio y circulatorio en el ámbito hospitalario.

**Tabla 2:** Pacientes (n = 162) según la presencia de lesiones e intervenciones asistenciales en la atención prehospitalaria e intrahospitalaria – Rio Branco, AC, Brasil, 2023.

Variables	Presencia de lesión		Valor de p
	No	Sí	
	n (%)	n (%)	
Inmovilización en el APH			
No	53 (43,1)	-	<0,001*
Sí	70 (56,9)	39 (100,0)	
Tipo de soporte			
Básico	97 (78,9)	32 (82,1)	0,668**
Avanzado	26 (21,1)	7 (17,9)	
Soporte respiratorio básico en el APH				
No	112 (91,1)	36 (92,3)	0,809**
Sí	11 (8,9)	3 (7,7)	
Asistencia respiratoria avanzada en el APH			
No	117 (95,1)	37 (94,9)	0,950**
Sí	6 (4,9)	2 (5,1)	
Soporte circulatorio básico en el servicio de urgencias			
No	97 (78,9)	23 (59,0)	0,014**
Sí	26 (21,1)	16 (41,0)	
Soporte circulatorio avanzado en el APH			
No	41 (33,3)	4 (10,3)	0,005**
Sí	82 (66,7)	35 (89,7)	
Asistencia respiratoria intrahospitalaria			
No	109 (88,6)	35 (92,1)	0,542**
Sí	14 (11,4)	3 (7,9)	
Apoyo circulatorio durante la hospitalización			
No	53 (43,1)	17 (43,6)	0,956**
Sí	70 (56,9)	22 (56,4)	

*Prueba exacta de Fisher;

**Prueba de chi-cuadrado de Pearson.

Leyenda: APH – Atención prehospitalaria.

Los datos de la Tabla 3 muestran que los grupos fueron similares en cuanto a la gravedad del traumatismo medida por los índices RTS en el ámbito prehospitalario (p = 0,480) y RTS (p = 0,404), REMS (p = 0,607) y mREMS (p = 0,839) en la fase hospitalaria, así como en el tiempo de permanencia en el servicio de urgencias (p = 0,265) y en la UCI (p = 0,561) y en el uso de ventilación mecánica (p = 0,108). Por otro lado, la duración de la hospitalización presentó una diferencia estadísticamente significativa entre los grupos (p < 0,001), siendo mayor entre los pacientes inmovilizados y con lesión (mediana de 3 días) en comparación con los que no presentaban lesión (0 días).

**Tabla 3:** Estadísticas descriptivas de los indicadores de la dimensión clínica según la presencia de lesiones – Rio Branco, AC, Brasil, 2023 (n = 162).

Variables	Presencia de lesión		p
	No	Sí	
	Mediana (Q1–Q3)		
Tiempo en urgencias (en horas)	2,6 (1,5 – 4,7)	2,0 (1,1 – 4,5)	0,265*
Tiempo en la UCI (en días)	14 (6 – 19)	14 (13,2 – 17,2)	0,561
Tiempo de ventilación mecánica (en días)	4 (3 – 6,5)	11 (9,5 – 12,5)	0,108
Duración de la hospitalización	0 (0 – 1)	3 (2 – 5)	<0,001**
RTS - APH	12 (12)	12 (12)	0,480*
RTS – Intrahospitalario	7,8 (7,8 – 7,8)	7,8 (7,8 – 7,8)	0,404*
REMS – Intrahospitalario	2 (0 – 4)	2 (0 – 3)	0,697
mREMS – Intrahospitalario	1 (0 – 2)	1 (0,2)	0,839

*Prueba de Wilcoxon-Mann-Whitney;

**Prueba de Brunner-Munzel.

Leyenda: UCI – Unidad de Cuidados Intensivos. RTS – Revised Trauma Score; REMS – Rapid Emergency Medicine Score; mREMS – modified Rapid Emergency Medicine Score.

El análisis de la asociación entre la presencia o ausencia de lesiones y las pruebas diagnósticas, las evaluaciones especializadas y las complicaciones clínicas puso de manifiesto diferencias significativas en algunas variables específicas (Tabla 4). En cuanto a las pruebas de imagen, la radiografía de miembros superiores fue más frecuente entre los pacientes inmovilizados con lesión (51,3 %) en comparación con los que no presentaban lesión (13,8 %) (p < 0,001). De manera similar, la radiografía de e e de las extremidades inferiores también se realizó de forma significativamente más frecuente en el grupo con lesión (69,2 %) (p < 0,001), lo que sugiere una mayor incidencia de lesiones ortopédicas en estos pacientes. En cuanto a las evaluaciones especializadas, la evaluación del traumatólogo (p < 0,001) fue más frecuente entre los pacientes inmovilizados con lesión (87,1 %) en comparación con los que no presentaban lesión (27,6 %).

**Tabla 4:** Pacientes (n = 162) según la presencia de lesiones y pruebas diagnósticas, evaluaciones especializadas y complicaciones clínicas – Rio Branco, AC, Brasil, 2023.

Variables	Presencia de lesión		Valor de p
	No	Sí	
	n (%)	n (%)	
Radiografía de tórax			
No	53 (43,1)	14 (35,9)	0,428*
Sí	70 (56,9)	25 (64,1)	
Radiografía de pelvis			
No	72 (58,5)	19 (48,7)	0,283*
Sí	51 (41,5)	20 (51,3)	
Radiografía de miembros superiores			
No	106 (86,2)	19 (48,7)	<0,001*
Sí	17 (13,8)	20 (51,3)	
Radiografía de las extremidades inferiores			
No	90 (73,2)	12 (30,8)	<0,001*
Sí	33 (26,8)	27 (69,2)	
Tomografía cervical			
No	95 (77,2)	31 (79,5)	0,769*
Sí	28 (22,8)	8 (20,5)	
Tomografía de columna			
No	99 (80,5)	33 (84,6)	0,564*
Sí	24 (19,5)	6 (15,4)	
Evaluación del traumatólogo			
No	89 (72,4)	5 (12,8)	<0,001*
Sí	34 (27,6)	34 (87,1)	
Valoración del cirujano vascular			
No	120 (97,6)	39 (100,0)	1,000**
Sí	3 (2,4)	-	
Valoración del neurocirujano			
No	75 (61,0)	26 (66,7)	0,524*
Sí	48 (39,0)	13 (33,3)	
Ingreso en la UCI			
No	116 (94,3)	35 (89,7)	0,325*
Sí	7 (5,7)	4 (10,3)	
Neumonía asociada a la VM			
No	122 (99,2)	37 (94,9)	0,144**
Sí	1 (0,8)	2 (5,1)	
Lesión por presión			
No	122 (99,2)	37 (94,9)	0,144**
Sí	1 (0,8)	2 (5,1)	
Infección del sitio quirúrgico			
No	122 (99,2)	37 (94,9)	0,144**
Sí	1 (0,8)	2 (5,1)	
Lesión renal aguda			
No	122 (99,2)	37 (94,9)	0,144**
Sí	1 (0,8)	2 (5,1)	

*Prueba de chi-cuadrado de Pearson;

**Prueba exacta de Fisher.

Leyenda: MMSS – Miembros superiores; MMII – Miembros inferiores; UTI – Unidad de terapia intensiva; VM – Ventilación mecánica.

En cuanto a las complicaciones clínicas (neumonía asociada a la ventilación mecánica, úlceras por presión, infección del sitio quirúrgico y lesión renal aguda), no se identificaron asociaciones estadísticamente significativas entre los grupos (p = 0,144). En general, estos resultados ponen de relieve que los pacientes inmovilizados y que presentaban lesiones presentaban afecciones asociadas al sistema musculoesquelético, como un mayor número de pruebas radiográficas de las extremidades y evaluaciones ortopédicas (Tabla 4).

Los datos de la Tabla 5 muestran una mayor frecuencia de alta hospitalaria tras la atención en urgencias entre los pacientes sin lesión (68,4 %) en comparación con los que presentaban una lesión (10,3 %). Por otro lado, los pacientes inmovilizados con lesiones presentaron un mayor derivación a unidades de mayor complejidad asistencial, destacando la unidad de ortopedia (33,3 %), el quirófano (23,1 %) y las unidades de hospitalización (23,1 %), lo que pone de manifiesto diferencias entre los grupos (p < 0,001).

**Tabla 5:** Pacientes (n = 162) según la presencia de lesiones y el destino tras la atención en urgencias y el desenlace final – Rio Branco, AC, Brasil, 2023.

Variables	Presencia de lesión		p*
	No	Sí	
	n (%)	n (%)	
Destino después de la urgencia			
Alta hospitalaria	84 (68,4)	4 (10,3)	
Unidad de ortopedia	11 (8,9)	13 (33,3)	<0,001
Quirófano	12 (9,8)	9 (23,1)	
Unidad de hospitalización	9 (7,3)	9 (23,1)	
Unidad de cuidados intensivos	4 (3,2)	3 (7,7)	
Evasión	1 (0,8)	-	
Unidad de observación	1 (0,8)	-	
Fallecimiento	-	1 (2,5)	
Otros	1 (0,8)	-	
Resultado final			
Alta hospitalaria	109 (94,8)	32 (84,2)	
Evasión	3 (2,6)	1 (2,6)	
Fallecimiento	2 (1,7)	3 (7,9)	0,044
Otros tipos de alta	1 (0,9)	-	
Traslado a otro hospital	-	1 (2,6)	
Alta a petición del paciente	-	1 (2,6)	

*Prueba exacta de Fisher.

En cuanto al resultado de la hospitalización (Tabla 5), también se observó una asociación estadísticamente significativa (p = 0,044) entre los grupos analizados en el estudio. El alta hospitalaria fue más frecuente entre los pacientes sin lesión (94,8 %) y la incidencia de fallecimiento entre aquellos con lesión (7,9 %). En general, estos hallazgos indican que los traumatizados inmovilizados y con lesiones presentan una elevada complejidad asistencial y un e a gravedad, evidenciadas por el derivación a sectores especializados tras la atención en urgencias y una mayor incidencia de desenlaces desfavorables, como el fallecimiento.

## DISCUSIÓN

Los resultados de este estudio ponen de manifiesto que la inmovilización en presencia de una lesión está directamente relacionada con las características del traumatismo, el perfil asistencial en la atención prehospitalaria y la evolución clínica de los pacientes. En cuanto al mecanismo del traumatismo, se observa que los eventos de mayor energía, como los accidentes de tránsito, se asociaron con mayor frecuencia a la inmovilización con lesión, lo que corrobora el hallazgo de un estudio^(17)^ que destaca estos eventos como los principales responsables de lesiones graves y potencialmente incapacitantes. Además, la mayor frecuencia de indicación en traumatismos con componentes mixtos (contusos/penetrantes) refuerza que la complejidad del mecanismo lesional influye directamente en la decisión clínica, tal como se discute en las directrices del *American College of Surgeons* ^(18)^, que enfatizan el mecanismo del traumatismo como uno de los pilares de la evaluación inicial.

En el contexto del APH, los resultados muestran una coherencia parcial (más del 50 % de los pacientes del estudio fueron inmovilizados y no presentaban lesiones) entre la evaluación clínica y la realización de la inmovilización, lo que indica que esta intervención sigue estando fuertemente incorporada a la práctica asistencial. La realización de inmovilizaciones en pacientes sin indicación clínica clara evidencia la persistencia de un modelo basado en la precaución universal. Este hallazgo es consistente con la literatura contemporánea^(18,19)^, que señala que la inmovilización se aplica con frecuencia de manera amplia, incluso en ausencia de evidencia sólida que respalde su uso indiscriminado. Las directrices recientes han recomendado la transición hacia estrategias basadas en el concepto de *Spinal Motion Restriction* (SMR), que orienta la restricción selectiva del movimiento de la columna a partir de criterios clínicos bien definidos^(9)^.

En este sentido, cuando no es posible descartar con certeza la presencia de una lesión, especialmente en situaciones de evaluación limitada en el servicio de urgencias, disminución del nivel de conciencia e intoxicación, la restricción del movimiento puede estar indicada como medida de precaución. Sin embargo, debe evitarse el uso indiscriminado de dispositivos rígidos, como la tabla larga, dando prioridad a enfoques más seguros, cómodos e individualizados. Así, la práctica actual no respalda la inmovilización rutinaria ante la incertidumbre, sino una toma de decisión clínica estructurada, que equilibre los riesgos y beneficios para el paciente^(9)^.

Además, la asociación observada entre la presencia de lesión y la mayor necesidad de soporte circulatorio en la atención prehospitalaria sugiere que estos pacientes presentan un mayor compromiso fisiológico y hemodinámico. Este hallazgo refuerza la relación entre la indicación de inmovilización y la gravedad clínica del traumatizado, mostrando que la intervención se ha empleado con mayor frecuencia en escenarios de mayor complejidad asistencial. Dicho resultado concuerda con la literatura, que demuestra que las intervenciones más avanzadas en el contexto prehospitalario tienden a concentrarse en pacientes con mayor gravedad del trauma y mayor riesgo de inestabilidad clínica^(20)^.

Así, la inmovilización en este contexto puede interpretarse como un marcador indirecto de gravedad, reflejando más la percepción clínica del riesgo por parte de los equipos de atención que una intervención que, por sí sola, determine los resultados. Esta interpretación refuerza que su indicación tiende a darse en pacientes con mayor compromiso fisiológico y potencial de inestabilidad.

En consonancia con estos hallazgos, la literatura señala que, en el escenario prehospitalario, el equipo de enfermería desempeña un papel central en la estabilización hemodinámica y clínica del paciente traumatizado, actuando en el soporte circulatorio y respiratorio mediante intervenciones como la punción venosa, la administración de medicamentos, el monitoreo continuo de los signos vitales, la administración de oxigenoterapia y la realización de vendajes^(21)^. En este sentido, la inmovilización debe entenderse como parte de un conjunto integrado de cuidados, que incluye también el mantenimiento de la perfusión tisular y la prevención de complicaciones secundarias.

En cuanto a la evolución clínica, se observó que los pacientes inmovilizados con lesiones presentaron un mayor tiempo de hospitalización, lo que refuerza su asociación con una mayor complejidad asistencial. Aunque las puntuaciones de gravedad no mostraron diferencias significativas entre los grupos, este hallazgo sugiere que la evaluación clínica inicial y el criterio profesional pueden identificar dimensiones de la gravedad que no se miden completamente mediante escalas fisiológicas. Este fenómeno es discutido por los investigadores^(22)^ al destacar que la toma de decisiones en traumatología es multifactorial, involucrando no solo parámetros objetivos, sino también el mecanismo del traumatismo, el contexto de la atención y la experiencia del profesional.

En el entorno intra-hospitalario, los pacientes inmovilizados con lesiones fueron sometidos con mayor frecuencia a exámenes diagnósticos y evaluaciones especializadas, especialmente en el área ortopédica. Este resultado refuerza la asociación entre la adecuación de la inmovilización y la sospecha de lesiones estructurales^(23)^. El mayor uso de exámenes de imagen en estos pacientes puede reflejar tanto una mayor gravedad como la necesidad de confirmación diagnóstica ante la posibilidad de lesiones ocultas, lo que evidencia un enfoque más cauteloso por parte de los equipos de atención.

En cuanto al destino tras la atención en urgencias y a los resultados hospitalarios, se observó que los pacientes inmovilizados y con lesiones presentaron una mayor necesidad de ingreso en unidades especializadas, una mayor frecuencia de intervenciones quirúrgicas y una mayor incidencia de fallecimientos. Estos hallazgos refuerzan la idea de que la inmovilización está fuertemente asociada a pacientes con mayor gravedad y complejidad clínica^(18,24)^. Sin embargo, es fundamental destacar que esta asociación no debe interpretarse como una relación causal. Evidencias recientes^(25,26)^ indican que la inmovilización prehospitalaria no está consistentemente asociada a la reducción de déficits neurológicos ni a la mejora de los resultados clínicos. Por lo tanto, la inmovilización debe entenderse como parte de un conjunto de intervenciones dirigidas a pacientes más graves, y no como un factor aislado determinante de la evolución clínica.

Ante este escenario, se hace evidente la necesidad de reevaluar las prácticas asistenciales relacionadas con la inmovilización en el trauma. La literatura apunta a la transición de un modelo basado en la inmovilización universal hacia un enfoque más selectivo, fundamentado en el concepto de *Spinal Motion Restriction (SMR).* Tal como se destaca en estudios y directrices^(9,27)^, este enfoque permite reducir intervenciones innecesarias, minimizar los riesgos asociados y mantener la seguridad del paciente, promoviendo una mayor racionalidad en la atención.

Así, los resultados de este estudio no solo refuerzan la asociación entre la inmovilización y una mayor gravedad clínica, sino que también evidencian lagunas en la adecuación de su indicación, destacando la importancia de incorporar protocolos clásicos basados en la evidencia, como los criterios del *National Emergency X-Radiography Utilization Study* (*NEXUS*) y la *Canadian C-Spine Rule* ^(28,29)^. La adopción de estas estrategias, junto con la capacitación continua de los equipos de salud, especialmente del personal de enfermería, que ocupa una posición estratégica en la evaluación y ejecución de estas intervenciones, puede contribuir a una atención más segura, eficiente y alineada con la mejor evidencia científica disponible.

Además, en cuanto a la inmovilización de las extremidades y la estabilización de la pelvis, se observa que estas intervenciones han recibido mayor respaldo en la literatura reciente en comparación con la inmovilización rutinaria de la columna. Los estudios indican que la inmovilización de las extremidades, especialmente en casos de fracturas sospechosas o confirmadas, contribuye a la reducción del dolor, la prevención de lesiones adicionales en los tejidos blandos y la disminución del riesgo de complicaciones durante el traslado del paciente^(30)^. En el mismo sentido, la estabilización pélvica temprana, mediante el uso de fajas pélvicas, se ha recomendado en pacientes con sospecha de fractura inestable de pelvis, y se asocia con la reducción del volumen pélvico, el control del sangrado y la mejora de la estabilidad hemodinámica^(30)^.

Las directrices de la *World Society of Emergency Surgery* refuerzan que la aplicación temprana de dispositivos de estabilización pélvica en la atención prehospitalaria y en la sala de emergencias constituye una medida importante en el manejo inicial del trauma pélvico grave^(30)^. Así, a diferencia de la inmovilización cervical indiscriminada, las inmovilizaciones de miembros y la estabilización pélvica presentan indicaciones mejor establecidas y respaldadas por la evidencia, por lo que deben incorporarse de manera criteriosa y temprana en los protocolos de atención, contribuyendo a la reducción de complicaciones y a la mejora de los resultados clínicos.

En resumen, los hallazgos de este estudio demuestran que la inmovilización con lesión se asocia a un perfil de pacientes con mayor complejidad asistencial en el contexto del trauma, evidenciado por la mayor frecuencia de traumatismos combinados, mayor necesidad de intervenciones circulatorias en la atención pre y de la realización de exámenes diagnósticos dirigidos al sistema musculoesquelético, mayor demanda de evaluación ortopédica y derivación a unidades de mayor densidad tecnológica, como el quirófano y la hospitalización. Además, estos pacientes presentaron un mayor tiempo de hospitalización y una mayor frecuencia de muerte hospitalaria, lo que indica que la adecuación de la inmovilización se relaciona más con la carga de lesiones y la complejidad de la atención que con la gravedad fisiológica medida por las puntuaciones tradicionales.

Por otro lado, la identificación de situaciones de inmovilización sin presencia de lesión, especialmente en pacientes con alta temprana de la sala de emergencias, pone de manifiesto debilidades en el proceso de toma de decisiones en la atención de primeros auxilios y indica que la toma de decisiones respecto a la inmovilización no ha estado plenamente orientada por criterios clínicos estructurados y basados en la evidencia.

Ante esto, los resultados de este estudio respaldan la necesidad de fortalecer las estrategias de educación continua y de implementar protocolos de atención basados en la evidencia, con énfasis en la evaluación clínica minuciosa, en el reconocimiento de lesiones potencialmente inestables y en la toma de decisiones orientada a la adecuación de la inmovilización a la condición clínica del paciente. En este contexto, deben priorizarse los enfoques selectivos, como la restricción del movimiento de la columna, y las intervenciones con mayor respaldo científico, como la inmovilización de las extremidades y la estabilización pélvica temprana.

Así, la calidad de la atención al paciente traumatizado requiere la integración entre el conocimiento científico actualizado, el razonamiento clínico calificado y la organización eficiente de los servicios de salud, con un enfoque en la adecuación de las intervenciones a la presencia de la lesión, lo que contribuye a la reducción de prácticas innecesarias, la optimización del uso de recursos y la mejora de los resultados clínicos.

Además, considerando la escasez de producciones científicas sobre el tema, se recomienda el desarrollo de estudios multicéntricos nacionales y prospectivos que permitan profundizar en la comprensión de la efectividad de las prácticas de inmovilización y sus implicaciones en los resultados clínicos de los pacientes traumatizados, especialmente en el ámbito de la atención de primeros auxilios.

### Limitaciones del Estudio

Entre las limitaciones de este estudio, cabe destacar que, aunque el hospital investigado constituye la principal unidad de referencia para la atención de traumatismos en el estado, la realización de la investigación en un único centro limita la generalización de los hallazgos a otros contextos asistenciales, especialmente aquellos con diferentes niveles de complejidad, organización de la red o perfil epidemiológico. El tamaño de la muestra del estudio también debe considerarse una limitación. Además, el uso de datos procedentes de los expedientes médicos de los pacientes para la identificación de las lesiones dependió de la calidad de los registros y de la exhaustividad de la información proporcionada por los profesionales.

### Contribución al Campo de la Enfermería, la Salud o las Políticas Públicas

Este estudio ofrece contribuciones relevantes para la práctica asistencial en la atención de primeros auxilios, particularmente en lo que se refiere a la inmovilización en caso de lesión y su relación con la evolución clínica y el pronóstico de los pacientes traumatizados. Los hallazgos evidencian una potencial aplicabilidad en la práctica clínica al apoyar al personal de enfermería en la identificación temprana de pacientes con mayor complejidad asistencial y mayor riesgo de desenlaces desfavorables, fortaleciendo el razonamiento clínico, la toma de decisiones y la priorización de la atención basada en criterios clínicos más precisos y alineados con las necesidades del paciente.

Además, al poner de manifiesto posibles situaciones de inadecuación de la inmovilización, los resultados refuerzan la necesidad de mejorar las prácticas asistenciales, con énfasis en la evaluación clínica minuciosa y en la adopción de intervenciones proporcionales al estado del paciente. En este sentido, el estudio contribuye al fortalecimiento de los enfoques basados en la evidencia y a la reducción de conductas potencialmente innecesarias o iatrogénicas.

Los hallazgos revisten especial relevancia en entornos marcados por limitaciones estructurales y operativas, como los observados en la Región Norte de Brasil, donde la adecuación de las intervenciones a la presencia de lesiones y la optimización de los recursos son determinantes para la calidad de la atención. En el ámbito de la gestión en salud, las evidencias generadas pueden respaldar la planificación, la organización y el perfeccionamiento de la Red de Atención a Urgencias y Emergencias, contribuyendo a la cualificación de las prácticas asistenciales, la estandarización de las conductas y el fortalecimiento de estrategias basadas en la evidencia en la atención a los traumatizados.

## CONCLUSIÓN

Los hallazgos de este estudio evidencian que la inmovilización con lesión en el contexto del trauma se asocia con características clínicas y asistenciales que reflejan una mayor complejidad de la atención, incluyendo mayor tiempo de hospitalización, necesidad de soporte circulatorio en el servicio de urgencias, realización de exámenes diagnósticos, especialmente orientados a la investigación de lesiones osteoarticulares, y derivación a unidades de mayor complejidad. Además, los pacientes inmovilizados que presentaban una lesión mostraron una mayor frecuencia de muerte.

La elevada proporción de situaciones de inmovilización inadecuada, evidenciada por el uso de la técnica y la ausencia de lesión, pone de manifiesto debilidades en la toma de decisiones en el servicio de urgencias, posiblemente relacionadas con la aplicación poco criteriosa de esta intervención. Este escenario refuerza la necesidad de reorientar los protocolos de atención, con énfasis en la evaluación clínica sistematizada, en el uso de herramientas de triaje validadas y en la incorporación de evidencia científica actualizada.

De este modo, se destaca el papel estratégico de la enfermería y del equipo multiprofesional en la mejora de la atención al traumatizado, especialmente en lo que se refiere al juicio clínico, la toma de decisiones y la garantía de que las intervenciones sean adecuadas a la presencia de lesiones. La implementación de procesos de formación continua, junto con el fortalecimiento de los sistemas de monitoreo y evaluación de la práctica asistencial, puede contribuir a la reducción de conductas innecesarias, la optimización de recursos y la mejora de los resultados clínicos en víctimas de trauma.

## Data Availability

Todo el conjunto de datos que respalda los resultados de este estudio se publicó en el propio artículo.
